# Courtship vocalizations of male mice do not always attract females

**DOI:** 10.1038/s41598-026-60012-z

**Published:** 2026-07-02

**Authors:** Maja Peng, Martina Zani, Reyhaneh Abbasi, Peter Balazs, Dustin J. Penn, Sarah M. Zala

**Affiliations:** 1 Department of Interdisciplinary Life Sciences, Konrad Lorenz Institute of Ethology, University of Veterinary Medicine, Vienna, Austria; 2 Acoustics Research Institute, Austrian Academy of Sciences, Vienna, Austria; 3 Vienna Doctoral School of Cognition, Behaviour and Neuroscience, University of Vienna, Vienna, Austria; 4 Acoustics, Analysis and AI, Interdisciplinary Transformation University Austria, Linz, Austria

**Keywords:** Ultrasonic vocalizations, Wild house mice, Playbacks, Preference, Y-maze, *Mus musculus musculus*, Ecology, Ecology, Evolution, Neuroscience, Zoology

## Abstract

Male house mice (*Mus musculus*) emit ultrasonic vocalizations (USVs) during courtship and mating, which influence their copulatory success. Several studies have found that playbacks of male USVs attract females, though this response may depend on the presence of male scent, and one study suggested that females are more attracted to complex than simple USV types. We conducted three playback experiments with wild-derived female house mice to test these predictions. None of our results met our expectations: First, females were not more attracted to sequences of complex versus simple USVs. Second, females were also not more attracted to these syllables when compared to silence. Third, females showed no attraction towards more natural sequences containing simple and complex USVs compared to silence, even in the presence of male scent. Thus, we found no evidence that wild-derived female mice show approach behavior towards male USVs, regardless of their complexity or exposure to male olfactory stimuli. These findings raise questions about the repeatability and generalizability of playback studies in house mice and highlight the need for more studies aimed at determining the types of vocalizations and contexts under which mice do and do not show attraction or other responses to USVs.

**Supplementary Information:**

The online version contains supplementary material available at https://doi.org/10.1038/s41598-026-60012-z.

## Introduction

The vocalizations of house mice (*Mus musculus*) and other rodents are mostly ultrasonic, outside the range of human hearing (> 20 kHz), and they can be surprisingly complex (laboratory mice:^1,2^; wild-derived *Mus musculus*^3,4^*:*). The ultrasonic vocalizations (USVs) of mice are emitted as variably discrete units that can be classified into different ‘syllable’ (or ‘call’) types, which include four to 15 different classes^1,3,5–11^. USVs are classified according to their spectro-temporal features, shape and complexity (e.g.^12^). Despite variation in the number and types of USV classes identified among researchers, they are invariably categorized into two main types: simple calls, composed of a single, fundamental element with a continuous or unbroken frequency track versus complex USVs, composed of discontinuous elements due to one or more abrupt frequency-jumps in the fundamental frequency track (laboratory mice:^1^; wild-derived house mice:^3,7,8,13^). During courtship, both sexes emit USVs, though males emit ca. 84–96% of these calls^14–16^. Male courtship USVs are emitted in bouts with temporal sequences and repeating phrases of syllables, similar to bird song^1^. The USV syllable repertoire emitted by mice changes over time and during courtship and mating: upon first sexual contact, mice emit mainly simple USVs, whereas during late courtship and mating they produce many more complex USVs (laboratory mice:^10,11,17,18^; wild-derived house mice:^6^). USV emission appears to facilitate male mating success, as certain features are correlated with and predictive of male copulatory and reproductive success^6,13,19^. It remains unclear, however, why house mice emit such a diversity of USV classes or whether mice can even discriminate between different types of USVs. Addressing these questions is necessary to determine their meaning (information transmitted) and signal functions (effects).

Female laboratory mice were found to be more attracted to vocalizing than non-vocalizing (experimentally silenced) males^20,21^, and many subsequent studies have tested the responses of female mice to male USVs using playback experiments (laboratory mice: e.g.^22–25^; wild-derived mice:^4,7,26^). Several studies have reported that females show approach behavior towards playbacks of male USVs (laboratory mice:^25,27,28^; wild-derived mice:^4,7,26^). Females are more attracted to playbacks of males of their own species than an unfamiliar *Mus* species^7^ and also to unfamiliar non-kin compared to familiar kin^4^. Yet, female attraction to male courtship USVs depends upon several factors, including their social experience, paternal exposure, and estrous stage^26^. Additionally, the physical presence of male mice or male odor seems to be crucial for sustaining a female’s attraction and interest in playbacks of male USVs^28^. Male odor (such as the male pheromone ESP1 or soiled male bedding) has been suggested to provide an ‘enhancing stimulus’ to increase or alter female attraction in playback studies^7,22,25^, though we are not aware of any experiments that have directly tested this hypothesis.

It is also unclear whether mice can discriminate among all types of USVs that males produce or whether they show preferential attraction towards certain call types. Laboratory mice can distinguish frequencies with only a 3% difference^29^ and can be trained to discriminate some types of USVs using an operant conditioning paradigm. In one study, mice (CBA/CaJ) were successfully trained to discriminate between simple versus complex USVs and between short versus long USVs^30^. In general, discrimination performance improved with more spectro-temporal dissimilarity of USVs. These findings align with a neurobiological study using the same stimuli, which showed distinct neural representations for different syllable types^31^. Further, Screven and Dent^32^ found that socially housed females learned to discriminate faster on average than isolated mice, and again, spectro-temporally dissimilar syllable types were easier to discriminate. Taken together, these studies show that mice can be trained to discriminate at least some USV syllable types. However, these results do not necessarily imply that mice discriminate USV types spontaneously, without intensive training. Operant conditioning assays are useful for assessing the perceptual limits of auditory discrimination, but they do not address whether untrained mice discriminate or show any preferences between different syllable types and, thus, whether different USV types might carry different information for the mice. These assays are also based on small sample sizes (e.g., n = 5^30^), as they are very time-consuming, and they do not investigate female attraction to male USVs.

Only one study to our knowledge has tested whether mice spontaneously discriminate among USV classes. Chabout and colleagues^33^ used a Y-maze assay to test the preference of female laboratory mice (B6D2F1/J, a hybrid of two inbred strains) towards playbacks of male USVs recorded in two different contexts: exposure to female urine versus a live female. The vocalizations recorded with the female urine stimulus contained a higher proportion of complex USVs, whereas the vocalizations recorded with the live female stimulus mainly consisted of simple syllable types. Females showed a preferential attraction for the playbacks containing a higher proportion of complex USV syllables. This is the only evidence to our knowledge that untrained mice can discriminate among USV classes and the only evidence that females prefer male courtship vocalizations containing more complex USVs. Wild house mice increase the complexity of their USVs during the course of courtship, and mice that emit more complex USVs and with larger repertoires tend to be more likely to copulate than other mice^6^.

We conducted three experiments with wild-derived house mice in which we presented females with recordings of male USVs to determine how they respond to playbacks in a Y-maze apparatus. First, we tested whether females spontaneously discriminate and show a preferential attraction towards complex over simple USV syllable types^33^. We used recordings of male courtship USVs and then experimentally manipulated the recordings to generate playbacks having either only complex or simple USVs. Second, to confirm that females show attraction to the USVs in our manipulated recordings, we tested their preferences for either simple or complex calls versus silence. We predicted that females would show attraction to both syllable types, though especially to complex USVs over silence. Third, we conducted a positive control and tested female attraction to recordings of more natural male courtship USV repertoire (or composition), consisting of multiple syllable types compared to silence. To test the hypothesis that female attraction to male USVs depends upon a male olfactory stimulus, we also compared females’ responses with and without an additional male olfactory stimulus. We tested females that were socially housed and mostly not in estrus, which are two factors that have been reported to increase female attraction to male USVs^26^.

## Methods

### Ethical statement

All experiments were conducted at the Konrad Lorenz Institute of Ethology, Austria, and in strict accordance with the Guide for the Care and Use of Laboratory Animals of the National Institutes of Health and the current Austrian laws. This study was approved by the Ethics and Animal Welfare Committee of the University of Veterinary Medicine, Vienna (Austria) (ETK-027/02/2021 and ETK-012/01/2025) in accordance with the University’s guidelines for Good Scientific Practice and current national legislation. The trapping of the parental wild mice was approved by the Ethics and Animal Welfare Committee of the University of Veterinary Medicine, Vienna in accordance with the University’s guidelines for Good Scientific Practice and authorized by the Austrian Federal Ministry of Education, Science and Research (ref BMBWF 2021-0.712.327) in accordance with current legislation. This study complies with the ARRIVE guidelines.

### Animals and housing

We conducted our experiments with wild-derived female house mice (*Mus musculus musculus*, n = 48, from 23 families), which were the F1-generation of wild-caught mice (herein, ‘wild mice’) originally trapped in seven different horse stables around Vienna and Lower Austria. We used wild-derived mice because results from one or a few laboratory strains do not necessarily generalize to wild mice, and because domesticated strains differ in their behavior from wild mice^34^. Moreover, the vocalizations of inbred laboratory mice differ from wild-derived strains^35^ and common laboratory strains suffer severe hearing loss at an early age, e.g., DBA/2 or C57BL/6 J;^36–39^). To create the F1 generation, we bred the mice between locations to control for inbreeding, as explained in^40^. We tested 18 females in experiment 1, 30 females in experiment 2, and 29 of these females were also used in experiment 3. We used recordings of 15 males (F2 generation, or F2) from 7 different families, and olfactory stimuli (scent marks) of 15 males (F6 generation of a previous wild-caught population^6,26,41^).

Mice were weaned on day 21 after birth and earmarked for individual identification. After weaning, they were kept in mixed-sex sibling groups until day 35, and then females were housed in groups of two to three individuals, while males were housed singly to avoid fighting. Due to our breeding procedure, some females were exposed to their father until weaning (usually the first litter), whereas others were never exposed to their fathers (usually the second litter). We have previously found that these rearing conditions did not affect females’ approaches to USV playbacks in a Y-maze^26^, and post-hoc analyses of the current dataset confirmed that paternal exposure did not affect the responses of these mice either (all *p* > 0.05, all η^2^ ≤ 0.08). Thus, we included females from both paternal rearing conditions (experiment 1: n = 9 females reared without father and n = 9 females reared with father; experiments 2 and 3: n = 14 and n = 13 females reared without father, respectively, and n = 16 females reared with father). All subjects were sexually mature but sexually inexperienced at the time of testing. Females were 334 ± 37 d (mean ± standard deviation (SD)) old (i.e., 11 ± 1.2 months; range: 9.4–12.9 months) when tested in experiment 1; 380 ± 34 d old (i.e., 12.5 ± 1.1 months; range: 9.7–13.6 months) in experiment 2; and 446 ± 35 d old (i.e., 14.7 ± 1.2 month; range: 11.9–15.7 months) in experiment 3. Note that wild-derived mice live longer and their fertility declines much later than in laboratory strains, as previously reported^42,43^. In our colony of wild-derived mice, females are routinely bred until 15 months of age without any noticeable decline in fertility, and our oldest reproducing female cohort ranged from 531 to 772 d old at the time of breeding. Females might breed even later, but we have not tried to breed older females. We also bred the population of mice used in the present study while the study was being conducted (including sisters of the females tested), and the oldest female breeder was 466 d old (15.3 months) at the time of fertilization.

None of the female subjects had previous experience with USV playbacks. From the 48 tested females, n = 17 were experimentally naïve and n = 31 were previously tested in a male odor preference experiment where they were exposed to scent marks for 15 min (i.e., experiment 1: n = 6 naïve, n = 12 with male odor experience; experiments 2 and 3: n = 11 naïve each, n = 19 (experiment 2) and n = 18 (experiment 3) with male odor experience). One female died after experiment 2 for unknown reasons.

We housed the mice under standard colony conditions with a 12:12-h light/dark cycle (dark period under red lights starting at 15:00 h in summertime and at 14:00 h in wintertime) with a room temperature of 21.31 ± 0.60 °C. Food and water were provided ad libitum. The tested mice were all kept in the same room and were not acoustically, visually or olfactorily isolated between cages. We kept our mice in a conventional open-cage husbandry, and we took care to have male and female cages in the same rack to expose all mice to odors of both sexes. We know from our experience in testing estrous stages and monitoring cycling that the females in our colony cycle under these conditions. All mice were first housed in standard type IIL cages (365 × 207x140 mm; Tecniplast, Germany) until 6–10 months of age, and then the entire colony was switched to standard type IIIH cages (425 × 266x185 mm; Tecniplast, Germany). Each cage contained aspen bedding (ABEDD, Austria), cotton nesting material (Plexx, Netherlands), a cardboard tube, a translucent red mouse house (Tecniplast, Germany) and a small wooden aspen gnawing block. Further environmental enrichment was provided in a 3-week alternating method (during each cage change): a running wheel (mouse igloo and Fast-trac, Plexx, Netherlands) or a hanging tube (Mouse Tunnel, Plexx, Netherlands). At each cage change, 15.5 g of seed mixture and five to ten dried meal worms were spread on the bedding as dietary enrichment.

### Experimental apparatus, design, and procedure

#### Y-maze apparatus

**Fig. 1:**
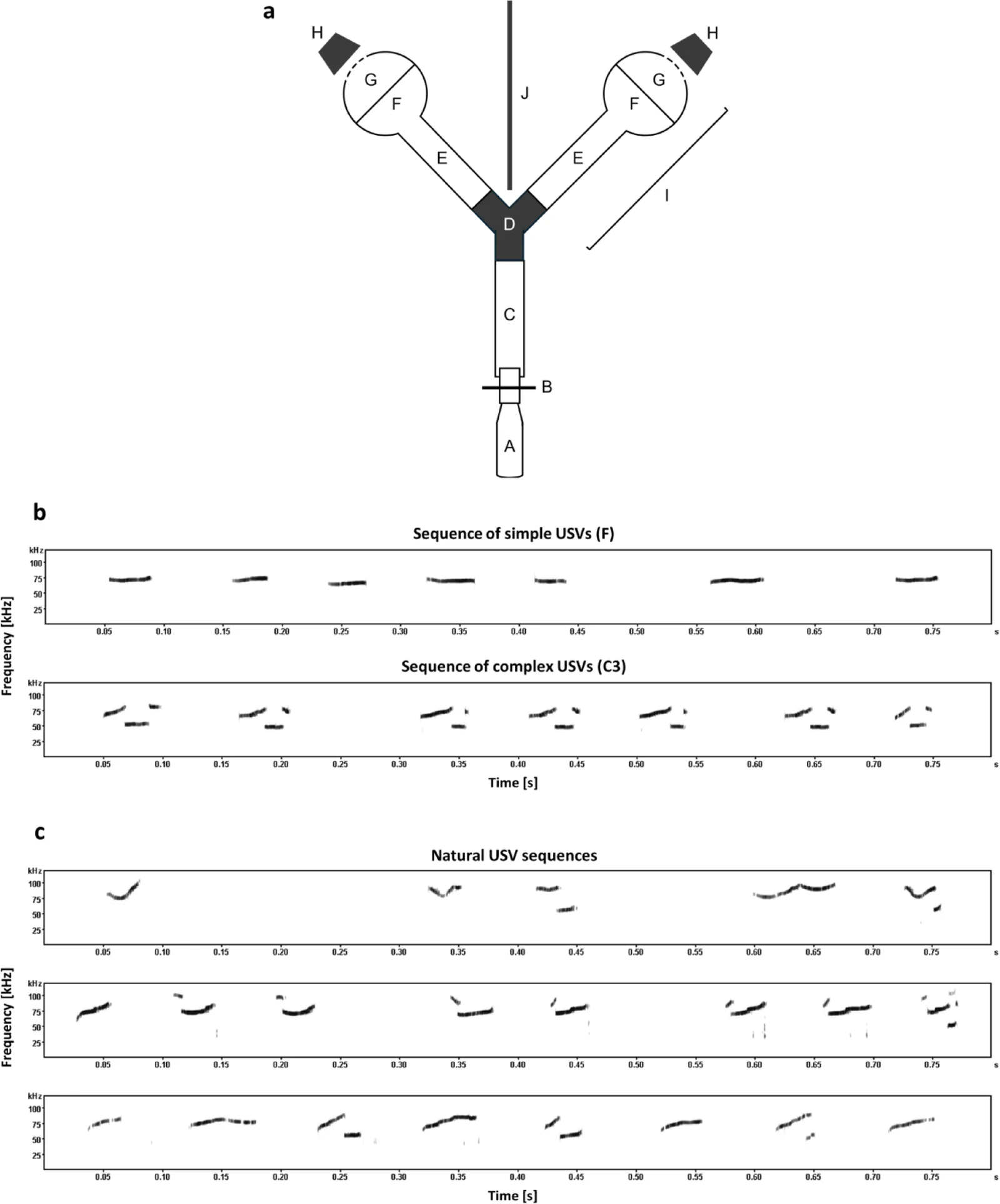
Diagram of apparatus and spectrographic examples of experimental playback stimuli. (**a**) Schematic of the Y-maze showing: A = handling bottle for transferring mice, B = gate, C = starting arm, D = intersection, E = stimulus arm, F = arena, G = speaker zone (arena includes speaker zone), H = speaker, I = stimulus side (stimulus arm and arena), and J = plexiglas wall (only used in experiment 3). Spectrograms of exemplary playback segments (0.8 s) of (**b**) the simple (F) and complex (C3) USVs used in experiment 1 and 2 (Supplementary Audio S1 and S2) and (**c**) the more natural USV sequences of three (out of n = 15) different male playbacks used in experiment 3. Note that these spectrograms of playbacks are short excerpts of the stimuli, and the complete spectro-temporal acoustic characteristics of playbacks are reported in the section “Generating audio playback files”.

The preference assays were conducted in a Y-maze (adapted from^26^ but we used longer arms to widen the amplitude gradient of the playbacks so that it is clearer from which side each playback is coming from), which was composed of three transparent plexiglas tubes (one for each stimulus arm and one for the starting arm of the Y-maze) connected by an opaque intersection in the middle (Fig. 1a). The starting arm (25 cm long, ⌀ = 5 cm) had a gate system at the entrance, i.e., a slit to allow a metal gate to be inserted into a 12.5 cm plexiglas tube (⌀ = 4 cm) for manual opening and closing of the entry to the Y-maze. Both stimulus arms of the Y-maze were 25 cm long (⌀ = 5 cm) and ended in a cylindrical arena (height = 8 cm, ⌀ = 16 cm). Arenas were covered with transparent plexiglas covers (19.5 × 19.5 cm) on the top and bottom to prevent the mouse from escaping. Each arena had one hole (⌀ = 5 cm) opposite the arena entry, which was covered with wire mesh. Approximately 5 cm behind each wire mesh, outside the arena, we placed ultrasonic speakers (Ultrasonic Dynamic Speaker Vifa, Avisoft Bioacoustics, Germany) attached to a power amplifier (UltraSoundGate Player 116H, Avisoft Bioacoustics, Germany) to project playbacks into the arenas and arms of the maze. The apparatus was lit from below with three infrared lights (48 LED Infrared Illuminator). We confirmed that the playback volume was within the amplitude range of male house mice vocalizations by manually comparing the amplitudes of the original recordings and our played back USV files in a spectrogram, while using the same microphone sensitivity as in the original recording. Throughout the apparatus, we also confirmed that there was an amplitude gradient by recording the playbacks with an ultrasound microphone (USG Electret Ultrasound Microphone, Knowles FG, Avisoft Bioacoustics) attached to an A/D converter (UltraSoundGate 416Hb, Avisoft Bioacoustics, Germany, 300 kHz sampling rate with 16-bit resolution) in different parts of the Y-maze. The playback was the loudest in the arenas and still detectable at the intersection of the Y-maze, whereas it was only faintly detectable in the starting arm. The volume was set on the third out of eleven gains for experiment 1 and 2 and on the fifth out of eleven gains in experiment 3. In experiment 1, where two stimuli were played simultaneously, both stimuli were matched as above at approximately equal volume in the intersection of the Y-maze. In experiment 3, an additional plexiglas wall was placed between the Y-maze arms to prevent any USVs from being detected in the silent arena (Fig. 1a).

Trials were video recorded with an overhead IP-camera (DCS-3710, D-Link Systems) using the software iSpy (64, version 7.2.6.0). Playbacks and videos were visually synchronized by screen recording (OBS Studio, 2024, version 29.1.3). The software RECORDER USGH-software (Avisoft-RECORDER, version 4.2) was used for playbacks. Playbacks were continually monitored (but not recorded) via ultrasound microphones (same as mentioned above) which were placed in front of the speakers to be sure that USVs were emitted from the speakers (as a positive control). Through monitoring we were also able to pick up potential vocalizations emitted by the females in the arenas. During all experiments, we detected almost no female vocalizations. Only 1–3 females across all experiments emitted 1 or 2 USVs. The females also did not squeak (broadband vocalizations). Audio and video monitoring were conducted outside of the experimental room to prevent disturbing the mice during the experiments.

#### Experiments

We performed three playback experiments, two of which were monomodal, using only USVs as a stimulus, and the third experiment was multimodal, using male odor as well as USVs. Below, we explain the details of each experiment and how we created the stimuli presented to the females.

*Experiment 1* We tested whether wild female mice respond differently to and, thus, discriminate between a complex (Complex 3, C3) versus a simple (Flat, F) syllable type. C3 syllables were defined as syllables < 91 kHz consisting of three elements separated by two frequency jumps. Flat syllable types were defined as syllables < 91 kHz with a frequency modulation < 5 kHz ^8,10,13^. Fs and C3s were selected because of their large differences in spectral features. Fs are characterized by minimal frequency modulation containing only one element, rendering them the simplest among the simple syllable types, while C3s contain multiple elements and frequency jumps. We played back Fs on one side of the Y-maze and C3s from the other side. The 18 females were tested during three testing days (i.e., six females per day). Sides of the playbacks were balanced among the mice and among testing days to avoid side bias.

*Experiment 2* To investigate whether females show attraction towards our manipulated playbacks, we tested whether they would approach (or avoid) the playbacks used in experiment 1 offering USVs on one of the Y-maze arms and silence on the other. Silence meant that no playback was played back from the speakers. This experiment consisted of two experimental groups, each testing 15 females (total n = 30). In one group, the Fs playback was played from one side of the Y-maze, while the other side remained silent. The other group was presented with the C3 playback on one side and silence on the other. Experiment 2 was conducted over four testing days. Individuals from the two groups were tested in alternating order within each day, and stimulus sides were counterbalanced across mice to control for order and side biases.

*Experiment 3* To confirm that females show an attraction to male USVs using more natural playbacks (positive control) we tested the preferences of the same females as in experiment 2 (n = 29). We also tested whether the presence of male odor (scent marks) influences female responses (multimodal approach), as suggested in the literature^7,22,44^. This experiment was conducted over four testing days. One female group (n = 15) was exposed to male odor, and the other group (n = 14) was tested without a male odor stimulus. We created 15 different playbacks from 15 different males to be used in the Y-maze (see below). In both experimental groups (with and without male scent marks), the USV playbacks were played from one side while the other side remained silent. The male scent marks were placed in both arenas of the maze. The sides of playback were systematically alternated and balanced among the mice, experimental groups and testing days, considering the number of USVs to avoid confounding effects of USV numbers between playback files.

#### Generating audio playback files

All USV playback files were created using Avisoft SASLab Pro (Avisoft Bioacoustics, Germany) and Audacity (version 3.5.1., Audacity Team, 2014). To create our playback files, we used recordings from a previous experiment. In short, 15 males (F2) were recorded while interacting with an unfamiliar and unrelated female behind a perforated plexiglas divider^8,40^. Males were previously stimulated with a female urine pool (15 µl) deposited on a filter paper one day prior to the recording to prime them^8^. Each female urine pool consisted of mixed urine of three females (F2). Stimulus females were also previously primed with a mixture of male soiled cage bedding (40 g, two weeks old) three days prior to the recording to induce estrous cycling. Each bedding mixture consisted of bedding from ten males (F2). On the recording day, males were placed inside one compartment, which contained a handful of the male’s own bedding and 15 µl urine of the stimulus female (deposited on a square of filter paper, 1.5 × 1.5 cm). Behind the perforated divider of the other compartment, we gently placed the stimulus female as well as a handful of her bedding. Males were audio-recorded for 10 min using ultrasound microphones (same as mentioned above), during which they could interact with the females through the divider.

Using these recordings, we created one playback file containing Fs and one containing C3s for experiment 1 and 2 (40 s long) (Fig. 1b, Supplementary Audio S1 and S2). To create the playback files, we first selected six different Fs and six different C3s from one male recording. Syllables were selected by visual inspection based on a high signal-to-noise-ratio. We aimed to avoid noisy signals (e.g. movement artifacts) and chose syllables with similar spectro-temporal features between Fs and C3s especially controlling for similar mean frequencies and amplitude (Fs: mean frequency = 69.2 ± 2.3 kHz, mean frequency bandwidth = 3.7 ± 0.8 kHz, mean amplitude = − 8.8 ± 8.8 decibels relative to full scale (dBFS); C3s: mean frequency = 64.1 ± 11.3 kHz, mean frequency bandwidth = 30.0 ± 2.7 kHz, mean amplitude = − 10.7 ± 8.2 dBFS). Then, all chosen syllables were high-passed at 40 kHz to cut most of the background (movement) noise which lies below this threshold and denoised with Avisoft SASLab Pro using the default settings (signals < − 60 dBFS were reduced by 90 dBFS) (Supplementary Fig. S1). The respective syllables were first ordered by length, numbered from 1 to 6, and then reassembled in a random order. The random list used was created with the sample function in R (Base R, version 2023.06.0 Build 421) using an equal probability for each syllable to be selected. Between each syllable we inserted silent inter-syllable intervals (ISI) by first creating six ISI that differed in length (min. = 49 ms, max. = 152 ms). We selected this range, as it is a typical distribution of ISI lengths observed in male–female courting contexts^6^. For these ISI we also created a random list in the same way as explained above. We then put the randomly ordered syllables and the ISIs together to create the playbacks. This meant, for example, that the first syllable in the syllable list was followed by the first ISI in the ISI list, followed by the second syllable in the syllable list and then the second ISI in the ISI list and so on. The same random lists were used for both playbacks (Fs and C3s) to ensure that both had the same rhythm. Both playback files were of equal length (40 s). Because Fs are generally shorter than C3 syllables (e.g.,^45^), the F file contained n = 292 syllables, whereas the C3 file contained n = 286 syllables. We chose a duration of 40 s for playbacks instead of a longer one, because in preliminary trials we found that when longer sequences of the same syllable type were played back to the mice, they started to move faster and jump. In addition, previous studies in laboratory and wild house mice have reported rapid habituation to the playbacks^4,23,27,28^.

In experiment 3, each female was presented with a different playback (and silence as control) (Fig. 1c). To create the more natural playbacks (180 s long) with a bigger repertoire of syllable types, we modified 15 recordings from 15 sexually stimulated wild males, recorded as explained above. Males’ recordings were chosen based on the highest number of emitted USVs. In each male recording, we used the first 180 s after the first vocalization was emitted, keeping the original sequence of syllables and bouts intact. We thus chose a longer playback duration compared to experiments 1 and 2, as these playbacks were not repetitive USVs as before, and represented natural USV sequences, including variation in syllable composition and repertoire. Long pauses lacking male USVs within each recording were cut to an inter-syllable or inter-bout length of at most 5 s, to ensure that females would be presented with consistent auditory stimuli. We applied high-pass filters from 20 to 50 kHz to remove frequencies in which the most background noise (e.g., from movement) occurred. Background noise that remained between USVs was manually replaced with silence from the same recording. Additionally, files were noise-reduced using the same approach used for the playbacks in experiment 1 and 2 (see above). The resulting modified playback files were 180 s long and with the first USV starting at 0.5 s. The final playback files contained on average 443 (SD = 139.6) syllables (min. = 276, max. = 816 syllables) with the following spectro-temporal features: mean frequency = 73.2 ± 6 kHz, mean frequency bandwidth = 22.3 ± 5.8 kHz, mean amplitude = − 14.1 ± 2.3 dBFS. Note that spectro-temporal features were extracted from the recording before the denoising step, as extracting these data after the denoising is not possible. Thus, all faint syllables are included in these features data, although some of them might have been removed by the denoising procedure. Playbacks were not normalized in their amplitude to reflect normal variation in amplitude inherent in courtship vocalizations.

#### Scent marks: Male olfactory stimuli

Scent marks used in experiment 3 were obtained from sexually mature wild males (F6). To collect scent marks, a blank filter paper (10 × 10 cm) was placed into the male home cages for 19 h overnight (17:00 to 12:00) with female urine (2 µl on a 0.5 × 0.5 cm scent mark paper) as stimulus. Each female urine stimulus consisted of a urine pool from three wild females (F1), which were all unrelated to the males. The filter paper containing the scent marks was retrieved and frozen at − 20 °C. We chose 15 different scent marked papers from 15 males to match the playbacks’ stimuli diversity. All scent marked papers were collected on the same date and were chosen through visual inspection selecting the highest rates of marking (see below for details). We cut a 4 × 4 cm square from these chosen filter papers selecting the highest marked area and even spacing of the scent marks. This square was then cut into two equal pieces (4 × 2 cm). The prepared odor stimuli were stored at − 20° C until a few minutes before each trial started. The scent marked filter papers used as stimuli had a mean coverage of ca. 46% (SD = 11.48). The area was determined by first photographing the stimuli under UV light and then using ImageJ (ImageJ, version 1.54 g, 2023) to convert them into binary images. The program GNU Image Manipulation Program (GIMP, version 2.10.32, 2024) was then used to calculate the number of pixels containing scent marks.

#### Testing procedure

The same procedure was applied to all three experiments. All experiments were conducted during the active period of the mice (between 14:30 and 18:00 h, under red light). We ensured that mice were tested no earlier than three days after their cage change. Before the trial, when odor had to be added (experiment 3), the two scent mark papers were placed in the middle of each arena. At the beginning of each trial, the female was transferred from her home cage to the Y-maze entrance using a handling bottle avoiding any direct contact. The handling bottle was placed in front of the trap door (Fig. 1a), which was opened to start the trial. Once the female voluntarily entered the Y-maze, the trap door was closed, and the experimenter left the experimental room turning off the red light. Only infrared light was used during testing. Before the USV playbacks started, females were given time to familiarize themselves with the apparatus. Specifically, mice needed to enter both arenas with all four paws twice to ensure that all mice had explored the whole maze before the experiment started. The median familiarization time for mice in all three experiments was 106 s (min. = 30 s, max. = 2216 s) (median given because only four mice took longer than 1000 s). After familiarization, playbacks were started once the female had moved back to the beginning of the starting arm (Fig. 1a). Experimental trials lasted for 40 s (experiment 1 and 2) or 180 s (experiment 3). At the end of each trial females were directed into the handling bottle and transported back into their home cages. After each trial, the set-up was exchanged for a clean one and cleaned with hot water. The trap door was cleaned with 70% ethanol. In experiments 2 and 3, a vaginal smear was taken from the females after the trial to assess their estrous stage^26^ to be able to statistically account for possible behavioral variation due to estrus. Estrous staging was conducted after rather than before the trial, to avoid possible influences on female behavior. Cells were examined under a light microscope (200 × magnification) and evaluated according to^46^. From experience in our laboratory, in wild mice it is not possible to assess the estrous stage by the vaginal opening and, despite cytological examinations, the prediction for estrus is not as reliable as in domesticated strains (personal observation). Proestrus and estrus were classified as “estrus” states, and metestrus and diestrus were classified as “non-estrus” states. For eight females (n = 2 in experiment 2 and n = 6 in experiment 3), estrous stage could not be determined due to low quantities of cells. In experiment 2, 18 females were in non-estrus and 10 were in estrus. In experiment 3, 18 females were in non-estrus and 5 were in estrus.

### Video processing and statistical analyses

Movement of the mice in the Y-maze was tracked automatically using EthoVision XT (version 17.5, Noldus). The center-point of the mouse was tracked during the familiarization time and the playbacks. During playbacks, behavioral tracking was done for the whole trial duration and also split into time bins of 20 s each. The zones of interest were defined as the arenas, the speaker zones, and the left and right sides of the Y-maze. The speaker zone was defined as the half of the arena in proximity to the speakers, and the arena zone included the speaker zone (Fig. 1a). The recorded variables were time spent in each zone (s), first entry into the zones, latency to first entry into the zones (s), and number of entries into each zone.

All statistical tests were conducted using R (version 2023.06.0 Build 421). All plots were created in R using the ‘ggplot2’ (version 3.5.1) and ‘ggpubr’ (version 0.6.0) packages. In experiment 1, one mouse had to be removed because of technical errors during the data collection, resulting in a final sample size of n = 17. No further mice had to be excluded for the main results. Side biases for all three experiments were analyzed using paired t-tests. First entries into the arenas were tested using binomial tests. Changes in time spent in the arena over the 20 s time bins were visually assessed and described by computing heatmaps. For all other analyses (i.e., time spent in the zones, entries and latency of first entry into each zone) linear mixed models were chosen to incorporate both fixed and random effects using the ‘lmer’ function from the package ‘lmerTest’ (version 3.1.3). The models were tested with the function ‘anova’ (‘stats’ package, Base R) with the Satterthwaite method for denominator degrees of freedom which provides more accurate *p*-values and confidence intervals when sample variances are unequal. If the chosen model contained interactions, type III ANOVAs were applied, whereas for models without interactions, type II ANOVAs were conducted. Results were considered statistically significant at a significance level of α = 0.05. Partial η^2^ were computed for effect sizes using the ‘effectsize’ package (version 0.8.9).

Fixed and random effects varied between the experiments and between main and exploratory results (Tables 1 and 2). In the first experiment, playback types (C3s or Fs) were used as fixed effects. The random effects (random intercepts) were mouse ID and testing day. However, testing day was dropped in all models in experiment 1 due to singular fit issues (i.e., its variance was estimated at (or near) zero) (described below). When we modelled ‘day’ effects (explorative analyses), testing day was used as an additional fixed effect while ID was kept as a random effect. In experiment 2, we combined the two experimental groups (Fs + C3s vs. silence) for modelling using stimulus type (playback or silence) and the playback type (C3s or Fs) as fixed effects. Estrous stage, ID and testing day were added as random effects. We chose estrous stage as a random effect because we wanted to account for it in our analyses as the literature is divided on the effects of estrus on female attraction to USVs^22,26–28^. Mice for which the estrous stage could not be determined were also included in the analyses (defined as “no cells”). In experiment 3, we merged the experimental groups (with and without odor); stimulus type (playback or silence) and odor treatment (with or without odor) were used as fixed effects. In addition to ID, estrous stage and testing day, playback and playback family (families of the recorded males) were included as nested random effects with the different playbacks nested within playback families because of the paired design (each playback was used twice) and because some playbacks of males belonged to the same families (Table 1).

**Table 1:** Overview of linear mixed-effects model outcome variables, fixed and random effects for each experiment (main results).

Experiment	Outcome variables	Fixed effects	Random effects	Notes
Experiment 1	Time spent in and number of entries into arenas and speaker zones, latency to first entry into arenas	Playback type (C3s vs. Fs)	ID, testing day (but see “Notes”)	Testing day was dropped in all models due to singular fit
Experiment 2		Stimulus type (playback vs. silence); playback type (C3s vs. Fs)	ID, testing day, estrous stage	In case of singular fit, estrous stage and/or testing day were dropped. ID and playback/playback family (nested) were never dropped due to the paired design
Experiment 3		Stimulus type (playback vs. silence); odor treatment (with vs. without odor)	ID, testing day, estrous stage, playback and playback family (playback nested within playback family)

**Table 2:** Overview of linear mixed-effects model outcome variables, fixed and random effects for each experiment (exploratory results).

Experiment	Outcome variables	Fixed Effects	Random Effects	Notes
Experiment 1	Time spent in arenas and speaker zones	Testing day; playback type (C3s vs. Fs)	ID	
	Absolute values of PPD of: 1. Total (ratio) data; 2. 40 s data In arenas and speaker zones	Phase preference (familiarization vs. playback); playback type (C3s vs. Fs)	ID, testing day (but see “Notes”)	Testing day was dropped in all models due to singular fit
Experiment 2	Absolute values of PPD of: 1. Total (ratio) data; 2. 40 s data In arenas and speaker zones	Phase preference (familiarization vs. playback); stimulus type (playback vs. silence); playback type (C3s vs. Fs)	ID, testing day, estrous stage	In case of singular fit, estrous stage and/or testing day were dropped. ID and playback/playback family (nested) were never dropped due to the paired design
Experiment 3	Absolute values of PPD of: Total (ratio) data in arenas and speaker zones	Phase preference (familiarization vs. playback); stimulus type (playback vs. silence); odor treatment (with vs. without odor)	ID, testing day, estrous stage, playback and playback family (playback nested within playback family)	

For all models, the simplest model of best fit was chosen based on a backwards stepwise model selection, as follows. First, possible interaction structures between fixed effects were visually evaluated (‘flexplot’ package, version 0.20.3). If interactions between fixed effects were not significant, they were dropped, and the simpler model was preferred. When model assumptions of normality or homogeneity of variance were violated, a Box-Cox transformation was applied (‘MASS’ package, version 7.3.60; ‘TeachingDemos’ package, version 2.13). If a Box-Cox transformation did not meet the model assumptions, square-root or logarithmic transformations were used instead based on which transformation met the model assumptions better. Model residuals and diagnostics were assessed using the DHARMa package (version 0.4.7 ^47^). If random effects had singular fit problems before the data transformation, we chose to drop the ones that were not inherent to the experimental design. This means that ID was always kept as a random effect despite potential singular fit problems in all experiments due to the paired experimental design. Playback and playback family in experiment 3 were also kept in the model despite occurring singular fit problems for the same reason. This means, only testing day and/or estrus were dropped in case of singular fit problems. However, in rare cases where the model could not properly converge, previously dropped random effects were put back into the model to solve the convergence issues.

In further explorative analyses, we investigated whether USVs caused any female behavioral differences comparing the familiarization and the playback phases in the Y-maze. As the familiarization and playback phase had different time lengths, we used two approaches to investigate this question. First, we compared both phases in their total duration (exploratory approach 1). We calculated and compared the relative time spent in each arena and speaker zone during the whole familiarization phase to the whole playback phase. We calculated this relative time by dividing the time spent in the zone of interest by the total time spent in the Y-maze for both phases. This was done for all three experiments. Second, we investigated female behavioral changes specifically at the onset of the playback and compared the total time that the mice spent in the zones of interest during the last 40 s of the familiarization phase with the playback phase (also 40 s) (exploratory approach 2). This analysis was done in experiment 1 and 2. In experiment 3 we did not compare the last part of the familiarization phase with the playback phase as the mice were retested and already familiar with the maze. Thus, the familiarization time was too short for comparison. Next, to investigate in which phase the mice spent the most time in the zones of interest, we calculated a “phase preference difference” (PPD) for both approaches. The PPD was the difference in (relative) time spent between the playback phase and the familiarization for each arena and speaker zone separately. Positive PPD values indicate that the female spent more time in the respective zone during the playback, and negative PPD values indicate that the female spent more time in the zone during the familiarization phase. The further the value is from 0, the stronger the preference for a phase. For modelling, we used the absolute values of the PPD as the outcome variable and added “phase preference” (familiarization or playback) as a fixed effect. The same modelling approach and all other fixed and random effects used in the main results described above were kept the same for these analyses (Table 2). Some mice had to be excluded during these last explorative analyses: In experiment 2, three mice were excluded from the total (ratio) data (exploratory approach 1) (final n = 27: n_C3_ = 13, n_F_ = 14) and 40 s data (exploratory approach 2) due to an overly long familiarization time (> 21 min). Additionally, in the 40 s data, some mice had no preferences, with PPD = 0 (either due to not entering the zones or due to spending the same time in the zones during the familiarization and playback phase), and thus, they were excluded from the analysis (final n_arena_ = 26, final n_speaker_ = 25). In experiment 3, one mouse was excluded from the total (ratio) data due to technical problems (final n = 28: n_withodor_ = 14, n_withoutodor_ = 14). Statistical outputs for all models are provided in Supplementary Tables S1–S7.

## Results

### Experiment 1: Do females prefer complex (C3) over simple (F) USVs?

**Fig. 2:**
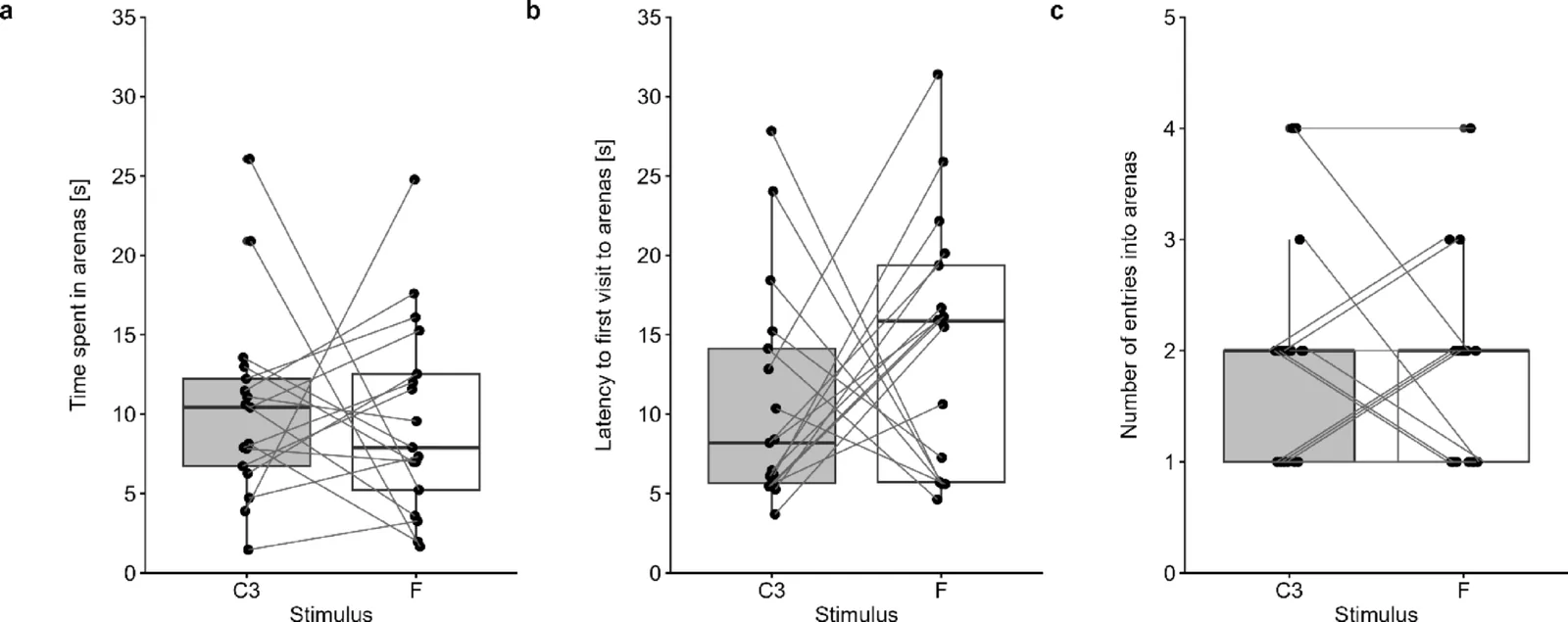
Female discrimination of playbacks of complex (C3) versus simple (F) syllables. Boxplots of (**a**) time [s] spent in the arenas, (**b**) latency [s] to the first visit into the arenas, and (**c**) number of entries into the arenas during the playbacks. Boxplots show the median, 25% and 75% quartiles and whiskers extending to the most extreme data points within 1.5 × the interquartile range. Dots represent individual data points. The same individuals are connected by lines, n = 17.

We first tested for a general side bias (time in s) in the Y-maze and confirmed that females did not have a preferred side in the Y-maze (negative control for our Y-maze apparatus) [t(16) = 0.01, *p* = 0.991]. We then tested preferences for syllable types. Females did not prefer one syllable type over the other: time spent in the speaker zones [F(1,32) = 0.27, *p* = 0.609, η^2^ = 0.00827] and time spent in the arenas [Fig. 2a; F(1,32) = 0.21, *p* = 0.648, η^2^ = 0.0066] did not differ. Further, at the descriptive level, no clear change over time was evident, i.e., the time spent in the arena over the two 20 s time bins (Supplementary Fig. S2). The females did not enter one speaker zone more than the other [F(1,32) = 0.11, *p* = 0.741, η^2^ = 0.00347], nor did they enter one arena more than the other [Fig. 2c; F(1,16) = 0.24, *p* = 0.632, η^2^ = 0.01]. First entries into the arenas did not differ (*p* = 0.33, n = 17), nor latencies to first enter the arenas [Fig. 2b; F(1,32) = 1.91, *p* = 0.176, η^2^ = 0.06]. Our explorative analyses investigated whether there was a day effect across testing days: neither the playback type [arena: F(1,14) = 0.13, *p* = 0.724, η^2^ = 0.00921; speaker zone: F(1,28) = 0.12, *p* = 0.73, η^2^ = 0.00433], nor the testing day [arena: F(2,14) = 0.62, *p* = 0.55, η^2^ = 0.08; speaker zone: F(2,28) = 0.8, *p* = 0.457, η^2^ = 0.05] influenced the time spent in the zones. However, there was a trend in the interaction between playback type and testing day in the arenas and speaker zones [arena: F(2,14) = 3.14, *p* = 0.075, η^2^ = 0.31; speaker zone: F(2,28) = 2.91, *p* = 0.071, η^2^ = 0.17] suggesting that preferences for playbacks might change depending on the testing day (Supplementary Fig. S3). Additionally, we compared the PPD of the time spent in the zones during the familiarization and playback phases. We found that the PPD of the relative time spent in the speaker zone between familiarization and playback was significantly different [Supplementary Fig. S4; F(1,31) = 5.90, *p* = 0.021, η^2^ = 0.16] regardless of the playback type (C3 vs. Fs) [F(1,31) = 1.26, *p* = 0.270, η^2^ = 0.04] showing that females preferred to spend more time in both speaker zones during playbacks than during the familiarization phase. We found no phase or playback type effects on PPD of relative time spent in the arenas, nor on PPD of time spent in the speaker zone and arena when comparing the last 40 s of the familiarization with the playback phase (Supplementary Table S3).

### Experiment 2: Do females prefer complex (C3) or simple (F) syllable types over silence?

We first checked side biases in the Y-maze apparatus and confirmed that female mice did not show any side bias in the time spent in each side of the Y-maze [t(29) = − 0.79, *p* = 0.43, n = 30]. We then tested whether females preferred USVs compared to silence and found that, overall, females did not avoid nor approach the playbacks more than silence: no effect of stimulus type (playback vs. silence) on time spent in the speaker zone [F(1,57) = 1.50, *p* = 0.226, η^2^ = 0.03]. The playback type (Fs or C3s) [F(1,57) = 0.0003, *p* = 0.987, η^2^ = 0.0000045] did not influence the time spent in the speaker zones either. Entries into the speaker zones did not differ between the playback and the silent speaker zone [F(1,29) = 0.004, *p* = 0.952, η^2^ = 0.000127], nor were they affected by playback type (Fs or C3s) [F(1,28) = 0.02, *p* = 0.901, η^2^ = 0.000562].

**Fig. 3:**
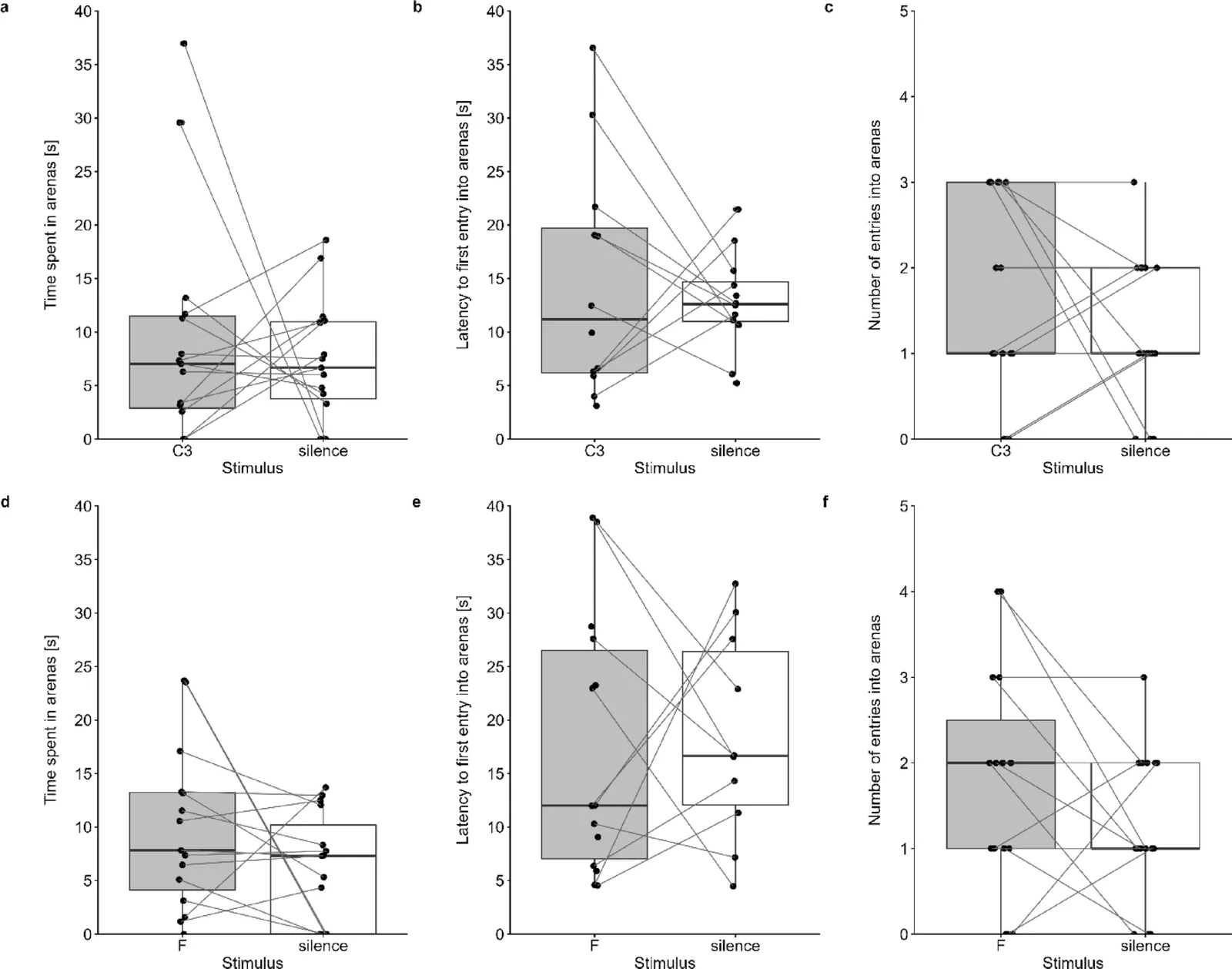
Female discrimination of playbacks (C3 or F) compared to silence. Boxplots of time [s] spent in the arenas, latency [s] to first visit to the arenas and number of entries into the arena for C3 vs. silence (**a**, **b**, **c**) and F vs. silence (**d**, **e**, **f**). Boxplots show the median, 25% and 75% quartiles and whiskers extending to the most extreme data points within 1.5 × the interquartile range. Dots represent individual data points. The same individuals are connected by lines. n_C3_ = 15, n_F_ = 15.

In the arenas, females did not spend more time in the playback compared to the silent arena [F(1,52) = 2.55, *p* = 0.117, η^2^ = 0.05], and playback type (Fs and C3s) did not influence females’ time either [F(1,41.609) = 0.0001, *p* = 0.994, η^2^ = 0.00000158] (Fig. 3a,d). At the descriptive level, no clear change was evident in time spent in the arenas over the two 20 s time bins (Supplementary Fig. S5). Entries into the arenas did not differ between the playback and the silent arena [F(1,29) = 1.92, *p* = 0.176, η^2^ = 0.06], nor were they affected by the playback type (Fs or C3s) [F(1,28) = 0.03, *p* = 0.866, η^2^ = 0.00104] (Fig. 3c,f). There was no difference between the first entries to the playback compared to silent arena in both experimental groups (C3s: n = 14, *p* = 0.79; Fs: n = 15, *p* = 0.30). Latencies to the first entry into the arenas did not differ between the playback versus silent arena [F(1,45) = 0.03, *p* = 0.872, η^2^ = 0.000588]. Playback type had no effect on the latencies either [F(1,45) = 2.11, *p* = 0.154, η^2^ = 0.04] (Fig. 3b,e).

Further exploratory analyses showed that females did not change their behavior (time) between the familiarization phase and the playback phase. Phase (familiarization vs. playback), stimulus (USV vs. silence) and playback type (Fs vs. C3s) did not affect the PPD of relative time spent in the speaker zones and arenas, nor the PPD of time spent in the two zones when comparing the last 40 s of the familiarization and the playback phase (Supplementary Table S5).

### Experiment 3: Do females approach male courtship USVs and is male odor necessary?

**Fig. 4:**
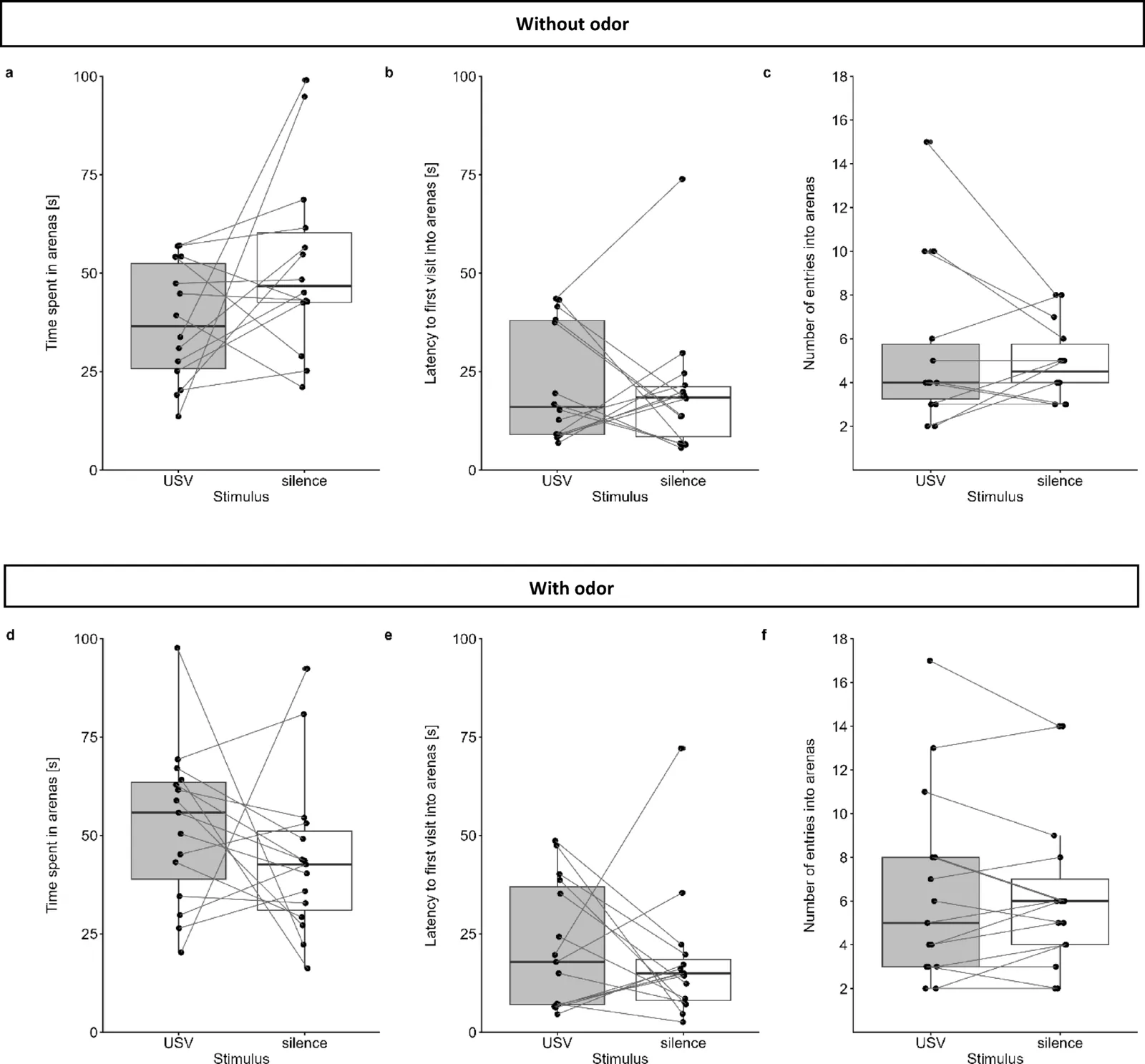
Female discrimination of more natural male USV sequences compared to silence with and without male odor. Boxplots of time [s] spent in the arenas, latency [s] to first visit to the arenas and number of entries into the arena without odor (**a**, **b**, **c**) and with odor (**d**, **e**, **f**). Boxplots show the median, 25% and 75% quartiles and whiskers extending to the most extreme data points within 1.5 × the interquartile range. Dots represent individual data points. The same individuals are connected by lines. n_without_ = 14, n_with_ = 15.

As before, we first checked side biases in the Y-maze apparatus and confirmed that females did not show any side bias in the time spent in each side of the Y-maze [t(28) = 0.29, *p* = 0.77, n = 29]. We then tested playback and odor effects and found that female mice showed no attraction towards a natural sequence of male USV playbacks (vs. silence) and that male scent marks did not affect this behavior: Time spent in the speaker zones, entries into speaker zones, time spent in the arenas (Fig. 4a,d), entries into arenas (Fig. 4c,f) and latencies to first entry into the arenas (Fig. 4b,e) did not differ (Table 3). Females’ first entries into the arenas did not differ either (without odor: n = 14, *p* = 1; with odor: n = 15, *p* = 1). At the descriptive level, no clear change in the time spent in the arenas over time (20 s time bins) was evident (Supplementary Fig. S6).

**Table 3:** Linear mixed-model results of stimulus type (USV vs. silence) and odor (with vs. without) effects on variables measured in the speaker zones and arenas of the Y-maze in experiment 3. F-tests using the Satterthwaite method are shown.

Predictor	Num df	Den df	F	*p*	η^2^
*Speaker zone*					
**Duration**					
(Intercept)					
Stimulus type	1	37.963	0.96	0.333	0.02
Odor	1	1.606	6.71	0.152	0.81
**Entries**					
(Intercept)					
Stimulus type	1	27.997	2.02	0.166	0.07
Odor	1	21.771	0.17	0.684	0.00775
*Arena*					
**Duration**					
(Intercept)					
Stimulus type	1	41.986	0.22	0.643	0.00516
Odor	1	41.993	0.15	0.699	0.0036
**Entries**					
(Intercept)					
Stimulus type	1	28	0.36	0.551	0.01
Odor	1	23.334	0.40	0.532	0.02
**Latency to first entry**					
(Intercept)					
Stimulus type	1	36.89	0.87	0.357	0.02
Odor	1	42.768	0.43	0.514	0.01

Again, we compared the familiarization with the playback phase and only found that odor treatment overall had a significant effect on the PPD of relative time spent in the speaker zone [Supplementary Fig. S7; F(1,52) = 7.51, *p* = 0.008, η^2^ = 0.13] but found no phase or stimulus type effect on PPD of relative time spent in the speaker zone [phase effect: F(1,52) = 1.33, *p* = 0.255, η^2^ = 0.02, stimulus type: F(1,52) = 0.94, *p* = 0.337, η^2^ = 0.02] implying that females preferred the speaker zones without odor more than with odor during both phases. We found no phase, odor or stimulus type effect on PPD of relative time spent in the arena (Supplementary Table S7).

## Discussion

We conducted three experiments with female wild-derived house mice to test their responses to playbacks of male courtship USVs. We found that: (1) Females showed no preferences between complex (C3) versus simple (F) syllables, contrary to our prediction based on a previous study^33^; (2) In the second experiment, females unexpectedly showed no attraction (or aversion) to these C3 and F playbacks versus silence. We therefore conducted an additional experiment to confirm female attraction to recordings of more natural male USVs, which contained a mixture of different types of USVs (positive control). (3) Females surprisingly showed no preferences for playbacks of male courtship USVs, regardless of whether male odor was present or not. Together, our results provide no evidence that wild-derived female mice show approach behavior towards playbacks of male courtship USVs, irrespective of USV complexity, syllable diversity, or the presence of male odor. To our knowledge, this is the first systematic playback study in house mice reporting a consistent absence of female approach responses across multiple USV stimuli and experimental contexts. Below, we discuss these findings and their implications, including results from our exploratory analyses.

### Experiment 1: Females did not prefer complex over simple USVs

Female mice did not show any preferences for the playbacks of complex (C3) compared to simple (F) USV syllable types, as none of our behavioral measures differed between the two stimuli. Thus, our findings do not support the suggestion that females show spontaneous preferences for playbacks of complex over simple USVs^33^. The differences in the results between the present and the previous study may be due to the mice or the playbacks used. We used wild rather than laboratory mice and we carefully controlled for potentially confounding spectro-temporal features by selecting syllables within similar frequency and amplitude ranges, resulting in playbacks with nearly identical mean frequencies and amplitudes. Further, the playbacks used by Chabout and colleagues^33^ included harmonic elements in the complex calls, which we did not include. Although we do not know how harmonic elements affect female behavioral responses, they seem to be strongly contextual and associated with male sexual behavior before mounting^6,10,48^. Their absence in our playbacks could have caused a different female behavioral response. Another difference concerns background noise: while we denoised the playbacks eliminating background noise, playbacks in the previous study included background noise in the ultrasonic range^33^. However, the behavioral effects of background noise and denoising in USVs playbacks remain unexplored and little can be said about their effects. While background noise might be disturbing, denoising might remove not just noise but other acoustic cues that naturally occur in live recordings. Taken together, differences in harmonic structure, acoustic context, or other uncontrolled features—rather than USV complexity per se—may underlie the contrasting results.

We also conducted several additional exploratory analyses. We tested potential testing day effects and found that female preferences for C3s versus Fs tended to change depending on the testing day (females tended to prefer Fs on the first, C3s on the second, and then showed no preference on the third day), though this effect was not statistically significant. To investigate whether the USV playbacks affected females’ behavior, we compared the familiarization phase to the subsequent test (playback) phase. We found that females spent more time (i.e., relative time) in the speaker zones during the playback phase than during the previous familiarization phase. This result indicates that females do not avoid USV playbacks and might even be attracted to them. However, because the familiarization phase necessarily preceded the playback phase, there is a potential time effect. For example, females could have spent more time in the speaker zones during the USVs playbacks because they became familiarized with the Y-maze, rather than being attracted to them. The observed pattern could thus reflect continued exploration with increased familiarity with the Y-maze rather than a response to the acoustic stimulus itself. This conservative interpretation is supported by the results of our comparison of the last 40 s of the familiarization to the playback phase (also 40 s). Here, we did not find any behavioral changes at the onset of the playbacks. Thus, the previous significant result in the speaker zones is most parsimoniously explained by a familiarization effect and these exploratory findings should be viewed with caution.

### Experiment 2: Females were not attracted to complex and simple USV playbacks compared to silence

We further investigated female attraction (or avoidance) to our manipulated recordings of male USVs by testing female responses to C3 and F playbacks compared to silence. Contrary to our expectations, we found no evidence for female attraction or aversion to USV playbacks, neither complex nor simple (versus silence), nor between the playback and the familiarization phases. One possible explanation for these results is that wild female mice may not respond to artificially manipulated playbacks (although studies in laboratory mice also use artificially manipulated playbacks (e.g.,^25,49^). Both of our playback types consisted of six repeated variants of a single syllable type. Such sequences, however, are unlikely to occur naturally during opposite-sex interactions as male USV sequences usually consist of multiple syllable types arranged in dynamic sequences that increase in diversity over time (i.e., the longer a sequence, the more syllable types occur)^6,13^. Although we intentionally introduced variation in our playbacks by constructing rhythmical ISIs, sequences only consisting of one syllable type might still be too artificial and might lack the ecological relevance to elicit female approach behavior. Further, our playbacks had similar mean frequencies and amplitude by design, and they might have lacked the dynamic changes in amplitude and spectral fluctuations that could be present in live vocal sequences. Thus, our experimental playbacks may have been too artificial to elicit female responses, including exposure to only one modality (auditory modality). Exposure to male odor during playback studies might be necessary, as previous studies have found female approach to USVs by using odor as an additional stimulus^7,22,25^. Hence, a multimodal sensory assay might be needed in playback studies, in addition to more natural syllable sequences. Therefore, we performed the third experiment.

### Experiment 3: Females were not attracted to more natural USV sequences and male odor did not influence their responses

We found no evidence that females were attracted to playbacks of more natural recordings of male courtship USVs for any of the measured variables, regardless of whether male odor was present or not. This result was unexpected, as Beck and colleagues^26^ found that wild females approached natural USV sequences in a Y-maze if they were socially housed and not in estrus. We mostly used the same procedures here, but there are some differences. First, Beck and colleagues sexually primed females two days before testing (by exposure to male bedding), which may have enhanced their motivation to investigate male USVs. Second, they left the background noise in the playbacks (and the control side), whereas we denoised the playbacks. Third, they shortened pauses between USVs to a maximum of 300 ms, while we chose a maximum of 5 s to keep a more natural rhythm in the USVs sequences. Fourth, Beck and colleagues used playbacks of a 40 s segment that was looped for 5 min, whereas the playbacks in the current study were natural USVs sequences of 3 min without looping. It is thus feasible that the higher vocalization rates in the previous study might have been more attractive to the females or, a non-exclusive alternative is that females avoided the background noise in the control side. It is also feasible that the denoising procedure in our study might have removed acoustic features, which are important to the females.

Another surprising finding is the absence of an odor effect, since male bedding as well as pheromones enhance preferences for USVs in laboratory mice^22^. Male odor cues are often used in playback studies with mice, either before the test trials as priming stimulus, as a treatment^22,41,44,50^ or as an enhancing stimulus^7,25^ to increase attraction to USVs and in almost all studies attraction to USVs could be observed. However, to our knowledge there have not been any playback studies using scent marks as olfactory stimuli. Another difference between some of these studies and our experiment is the position of odor. We placed both scent mark papers close to the speakers in the arenas, while in other studies only one odor stimulus was placed at the beginning of the testing apparatus in a neutral zone (e.g.^7,22^), which might interfere less with the approach behavior to the speakers (but see^25^). However, our exploratory analyses found that male odor had an overall negative effect on the PPD in the speaker zones, with females spending less time in the speaker zone when odor was present compared to when odor was absent, both in the familiarization and playback phase. Interestingly, there was no odor effect on the PPD regarding the entire arenas. This discrepancy could be explained by the placement of the scent mark paper, which we placed in the middle of the arenas, but outside the speaker zones. Females may have spent more time investigating the odor source itself rather than the speakers, leading to reduced time in the speaker zones, but not in the arenas. However, because these analyses were exploratory, this interpretation remains speculative.

In all three experiments we did not find any behavioral biases in the mice’s first entries into the arenas. We can exclude the possibility that the mice had difficulties in localizing the source of playbacks within the Y-maze because we checked the amplitude gradients of all playbacks. In contrast to previous research^25–28^, females’ time spent exploring the arenas of the Y-maze seemed to be constant over time, with females neither gaining nor losing interest in USVs over the duration of the trials. This finding suggests that females did not find the USV playbacks aversive. The consistently negative results in the present study contrast previous studies as, to our knowledge, all published behavioral USV playback preference studies (13 studies) report attraction of female mice to male USVs^4,7,22,23,25–28,33,35,44,49,51^. It is possible that other factors could have affected our results. For example, since the speakers were in fixed positions, playbacks lose interaction-dependent and spatial cues that live calls might have which might make mice less likely to approach.

Our mice were 11–14 months old, and though age might explain our negative results, this is doubtful for the following reasons. First, we routinely and successfully breed wild-derived females until 15 months of age. Thus, we have no reasons to suspect that the females used in this study were near reproductive senescence, had decreased ovarian hormones, or were less sexually motivated than younger mice. Second, many laboratory strains show early onset hearing loss, but we are not aware of any evidence of early hearing loss in wild house mice. Ralls^39^ conducted the only audiometric study in wild house mice to our knowledge, and found that they have a greater sensitivity to high frequencies (Hz) than laboratory mice (DBA/2 J and BALB/cJ), though the ages of the wild mice were not known. Third, previous USV playback studies with wild-derived house mice successfully used mice of following ages: 306 ± 169 d ^4^ , 209 ± 72 d ^7^, and 202–332 d ^26^. Thus, we have no reason to suspect that the age of our mice explains our negative findings, but future studies should consider age as a potential factor.

Another important factor might be the females’ estrous stage, as previous studies in laboratory mice^22^ as well as a study in wild mice^26^ found a greater preference towards USVs in non-estrous females. Compared to Beck and colleagues^26^, which had a higher proportion of estrous females, in the present study, most of the females were non-estrus (experiment 2: 18 in non-estrus, 10 in estrus; experiment 3: 18 in non-estrus, 5 in estrus). This may be due to the sexual priming used in^26^. In the present study, we found no evidence that the estrous stage of the mice influenced the results (see Supplementary Fig. S8 and S9), though, with the high ratio of non-estrus females, we expected a stronger preference towards USVs, not a lack of preference. Thus, our study does not support previous evidence for a non-estrus effect on USV attraction. Other studies on laboratory mice also reported mixed results on the effects of estrus on female attraction to male USVs^22,27,28^. However, our findings should be viewed with caution, as they are descriptive since estrus was not experimentally manipulated leading to uneven sample sizes. We only measured the estrous stage after the trials to avoid affecting females’ behavior before the experiments. Thus, the estrous stage was only included as a random effect to account for potential behavioral variability. More studies that directly test the effect of estrous stage on female responses to male USVs are needed.

The discrepancies between the present and previous studies (partly in our own laboratory) raise several questions. Results from playback studies in laboratory and wild mice might not be as robust as previously assumed. These discrepancies could have been caused by numerous differences in methodology (mice, sample sizes, differences in playback systems and stimuli preparation, such as acoustic preprocessing), which underscore the challenge in addressing the ongoing replication crisis in science. Since USVs do not travel beyond 2 m ^52^ and courtship USVs are mostly emitted during close social contact (e.g.^6,53^), it is clear that their function is not to call or attract females from far away. However, our results—combined with a study that found no evidence that females alter their movements (acceleration) while males are vocalizing during opposite-sex interactions^54^—raise the question of whether the function of male courtship USVs is to ‘call’ females to approach them, and strongly suggest that alternative functions need more attention (e.g., inducing estrous cycling and sexual receptivity^41^). It is also possible that USV playbacks are too artificial as, for example, male mice do not normally produce repeated loops of the same song (unlike some songbirds) and playbacks have not been tried that interactively respond to females’ vocal emissions or other behaviors (although we detected almost no vocal response by the females in our trials through audio monitoring). Thus, more behaviorally complex studies regarding the function of courtship USVs in mice might be useful and we encourage future studies to provide detailed methodological, and comprehensive, complete descriptions of each experimental step to enable replication over and within different contexts and laboratories^55^.

## Conclusions: Limitations and future directions

Our study is the first to experimentally test spontaneous USV syllable discrimination in house mice, and we point out some caveats and future directions. Our results do not imply that wild mice cannot discriminate between simple and complex USVs, nor that they do not show attraction for natural sequences of male USVs; but they suggest that another motivational state or context (perhaps even operant conditioning) might be required for wild female mice to show such responses. We tested females that were socially housed and mostly not in estrus, the two factors reported to increase the attraction of wild female mice to male USVs^26^, yet there might be other factors explaining our negative results (e.g., sexual priming with male scent). Further, our findings do not rule out the possibility that male USVs induce female approach to vocalizing males^20,21^; nor do they rule out the hypothesis that male USVs, complex or otherwise, have other influences on females, such as estrous cycling^41^ or receptivity to male copulatory behavior^6^. Nevertheless, our findings challenge the hypothesis that females are generally attracted to USVs, as our females showed no preference for male courtship USVs (F, C3, or more natural USV sequences) over silence, suggesting that the conditions under which mice respond to playbacks of USVs might be even more limited than previously reported^26^. They also raise questions about the repeatability of playback experiments in mice. Overall, our results highlight the need for rigorous testing of assumptions regarding USV perception in mice, particularly the assumption that male USVs are inherently and universally attractive to females. Therefore, more studies involving different types of vocalizations, subjects and playback assays are needed to better understand the conditions under which mice do and do not discriminate or show attraction to different vocalizations (e.g., tests of subjects of different ages, sexual experience, stages of estrus in response to different USVs and other vocalizations^17^). Female mice may mainly show attraction towards USVs when a male mouse is present or engaged in sniffing or other courtship behaviors. Future studies should systematically manipulate acoustic structure, behavioral context, and multimodal cues using more ecologically relevant stimuli to better understand when—and whether—female mice respond to USV playbacks.

## Supplementary Information

Below is the link to the electronic supplementary material.

Supplementary Material 1

Supplementary Material 2

Supplementary Material 3

## Data Availability

The datasets generated and analyzed during the current study are available in the repository of the University of Veterinary Medicine Vienna (Phaidra) via the following link: [https://phaidra.vetmeduni.ac.at/o:5001] (https://phaidra.vetmeduni.ac.at/o:5001).

## Abbreviations

C3: Complex 3 (syllable type)
F: Flat (syllable type)
ISI: Inter-syllable interval
PPD: Phase preference difference
USV: Ultrasonic vocalization
