# Supplementary material for: Courtship vocalizations of male mice do not always attract females
Source: Sci Rep. 2026 Jul 2;16:30398. doi: 10.1038/s41598-026-60012-z (PMC13630556; doi:10.1038/s41598-026-60012-z)
Supplement: Supplementary file 1 [file 41598_2026_60012_MOESM3_ESM.pdf]

Courtship vocalizations of male mice do not always attract females

Maja Peng, Martina Zani, Reyhaneh Abbasi, Peter Balazs, Dustin J. Penn, Sarah M. Zala

Supplementary audio (captions):

Supplementary Audio S1: F playback used in experiment 1 and 2. The file contains a cropped excerpt of the original recording, which is time-expanded by a factor of 15 using a time-scale conversion to make the ultrasonic signal audible.

Supplementary Audio S2: C3 playback used in experiment 1 and 2. The file contains a cropped excerpt of the original recording, which is time-expanded by a factor of 15 using a time-scale conversion to make the ultrasonic signal audible.

Supplementary figures:

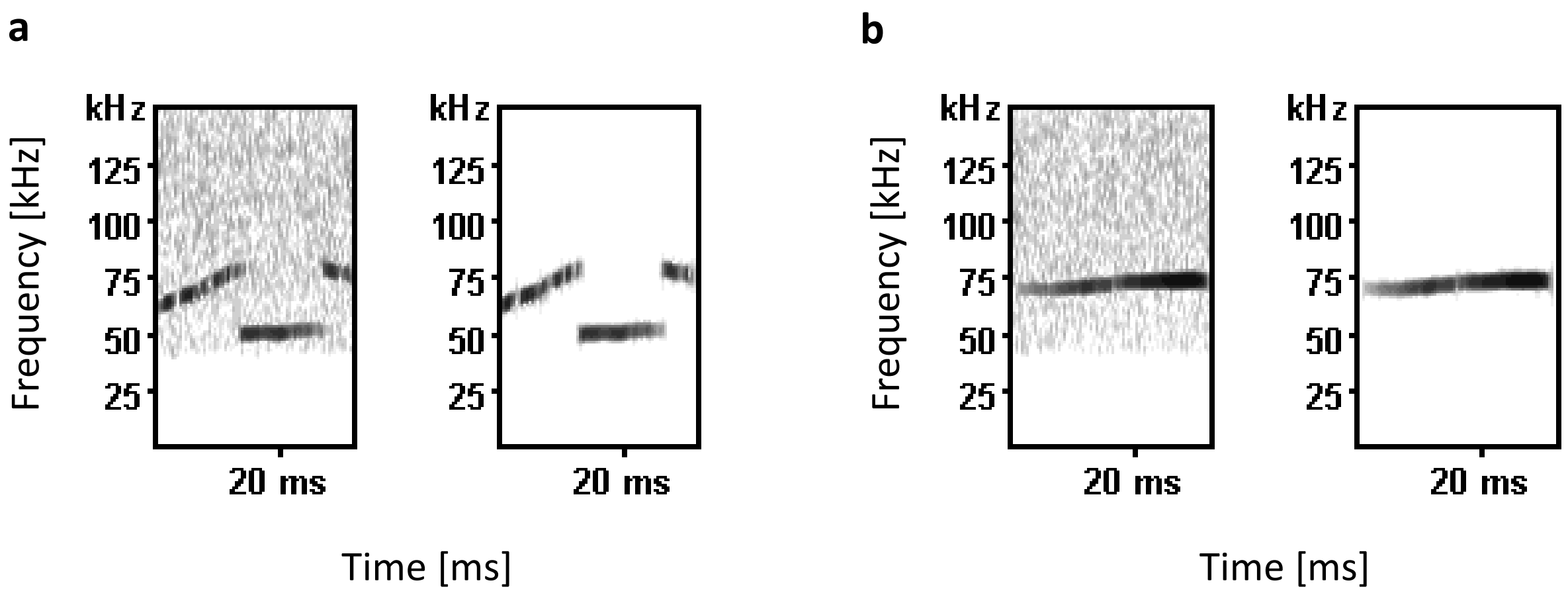

Supplementary Figure S1: Spectrograms of noisy and denoised USV syllables. a) C3 syllable (one of the six syllables used in the C3 playback), b) F syllable (one of the six syllables used in the C3 playback). Noisy spectrograms were already high-passed at 40 kHz. Denoising was done in Avisoft SASLab Pro using the default settings (signals <-60 dBFS were reduced by 90 dBFS).

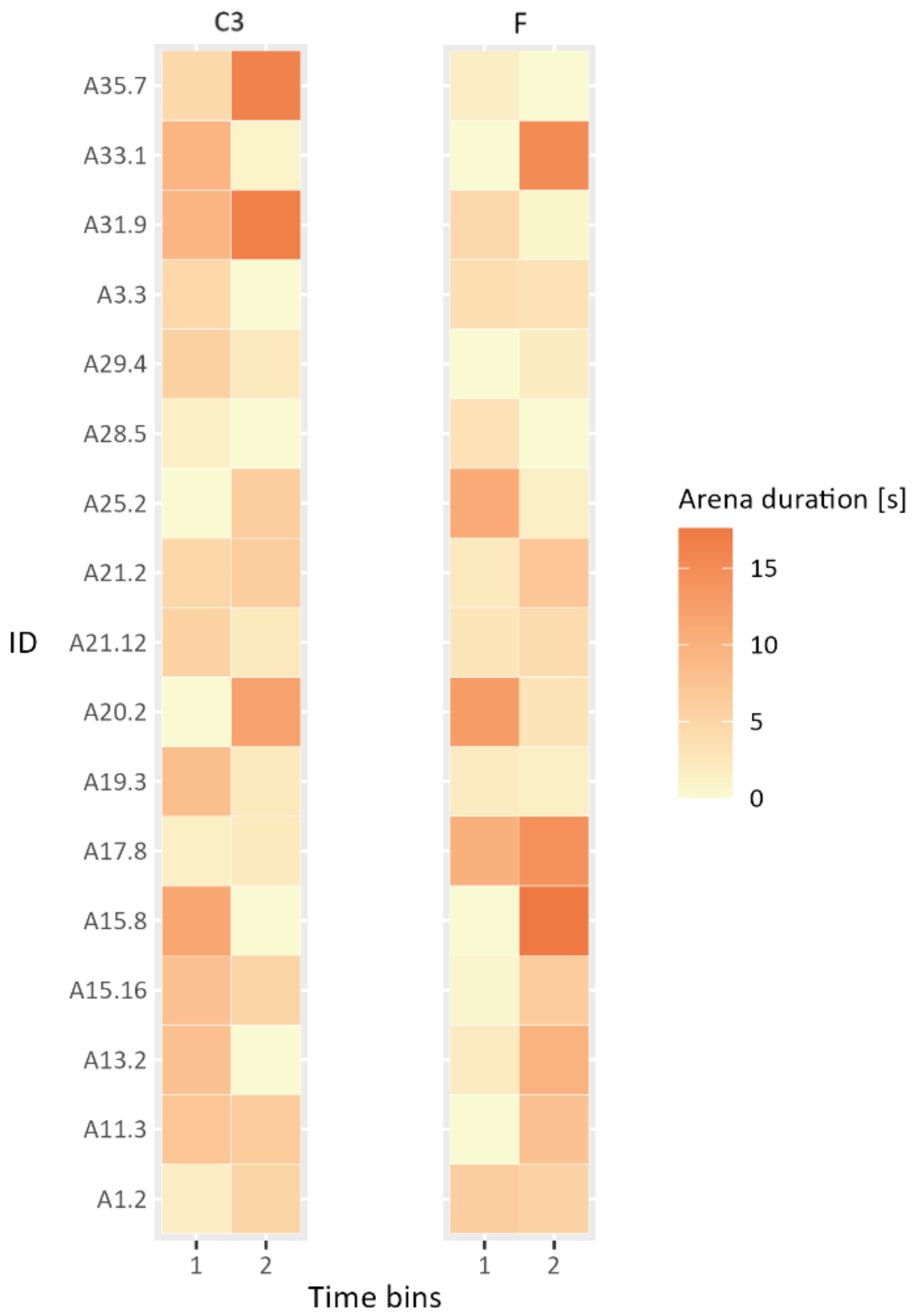

Supplementary Figure S2: Time spent in the C3 versus F arena per individual ID (n=17) in experiment 1. The time is split into two-time bins of 20 s each.

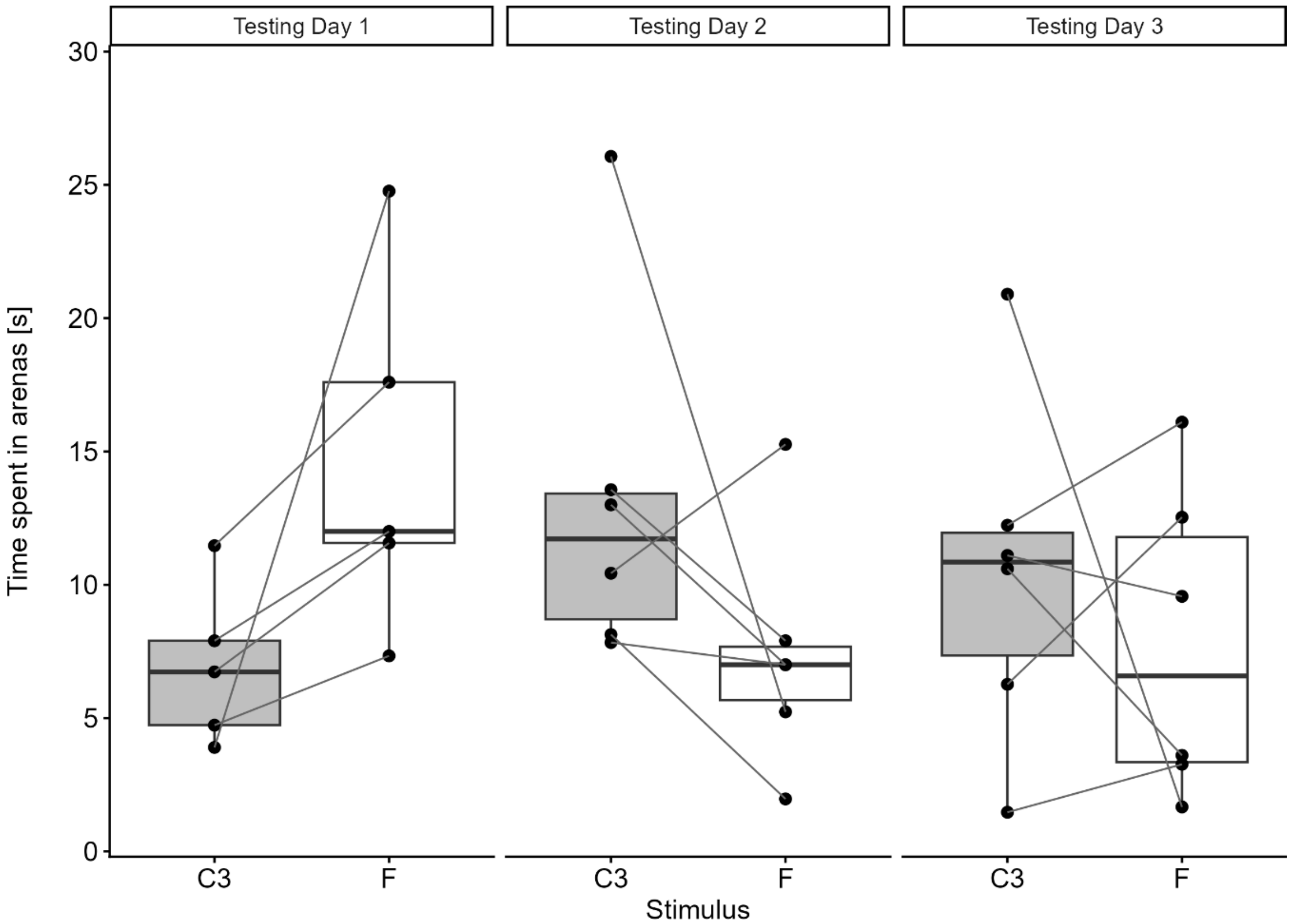

Supplementary Figure S3: Day effects in behavioral responses to C3 vs. F syllable playbacks. Boxplots of time spent in the arenas are shown. Boxplots show the median, 25% and 75% quartiles and whiskers extending to the most extreme data points within 1.5x the interquartile range. Dots represent individual data points. The same individuals are connected by lines; n_{day1}=5, n_{day2}=6, n_{day3}=6.

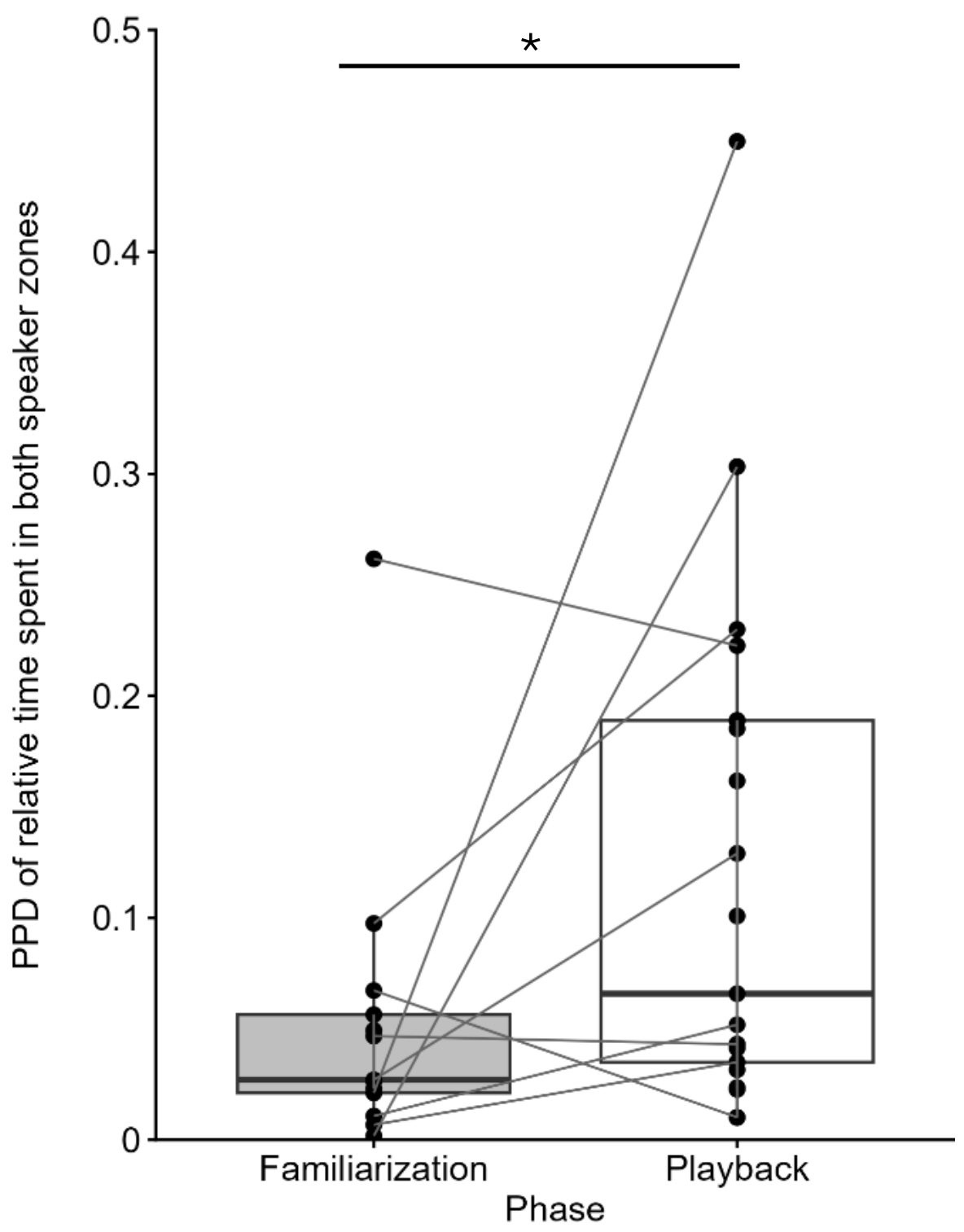

Supplementary Figure S4: Phase preference in experiment 1. Boxplot of phase effect (familiarization vs. playback) on the phase preference difference (PPD) of relative time spent in the speaker zone. Boxplots show the median, 25% and 75% quartiles and whiskers extending to the most extreme data points within 1.5x the interquartile range. Dots represent individual data points. The same individuals are connected by lines; n=17; *=p≤0.05.

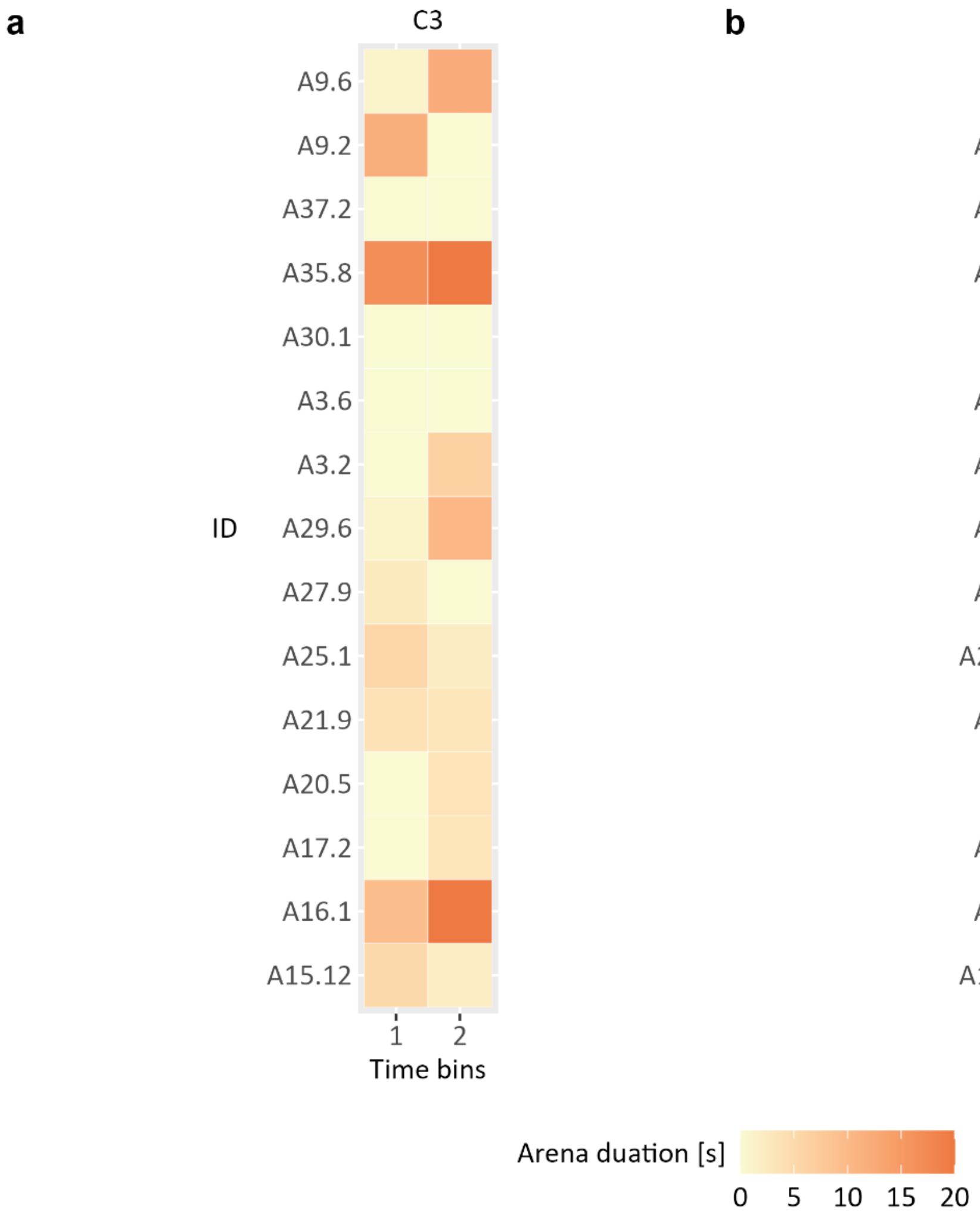

Supplementary Figure S5: Time spent in the a) C3 arena and b) F arena per individual ID (n_{C3}=15, n_F=15) in experiment 2. The time is split into two-time bins of 20 s each.

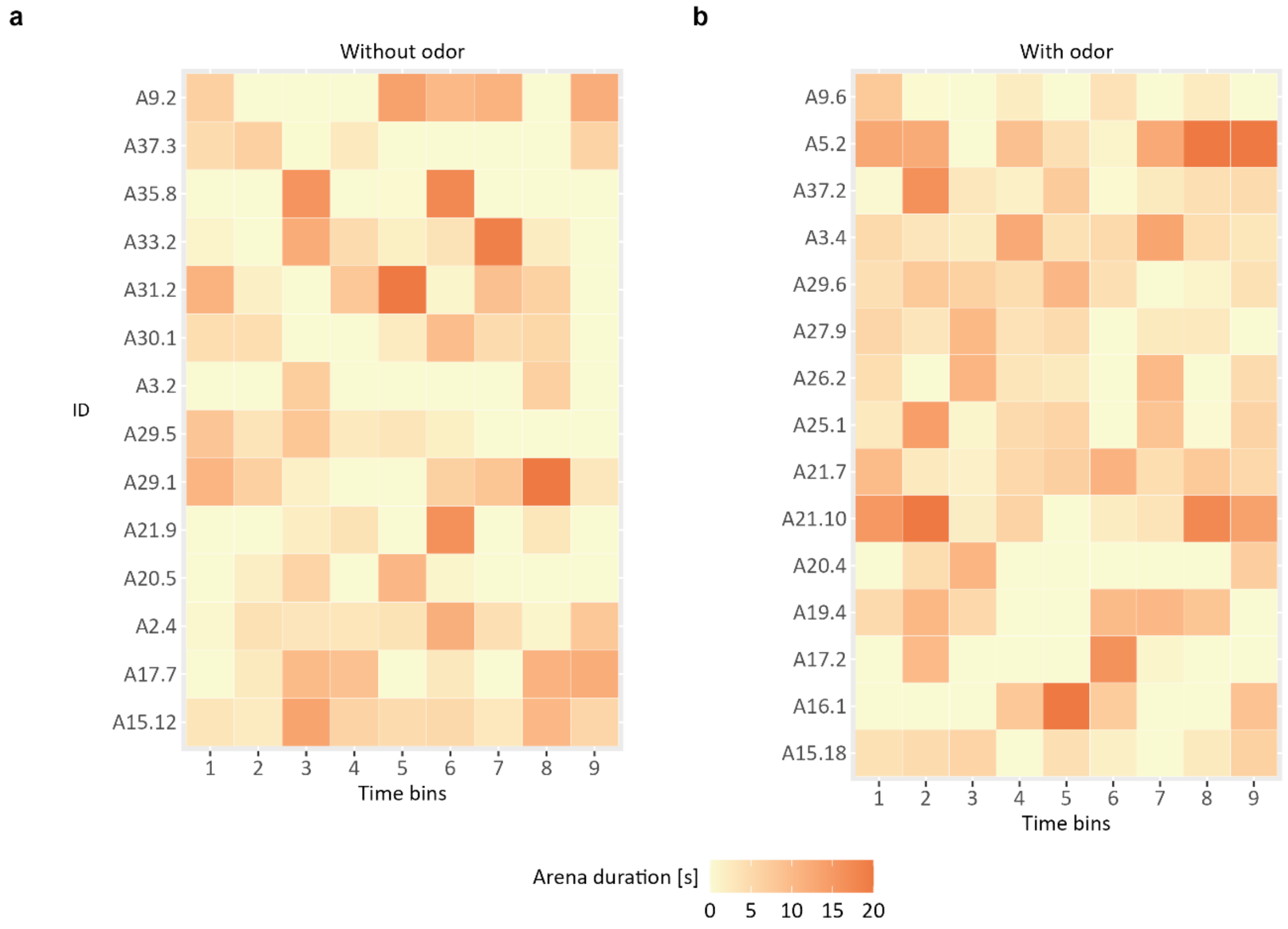

Supplementary Figure S6: Time spent in the USV playback arena for a) without odor and b) with odor treatment per individual ID ($n_{without}$=14, n_{with}=15) in experiment 3. The time is split into nine-time bins of 20 s each.

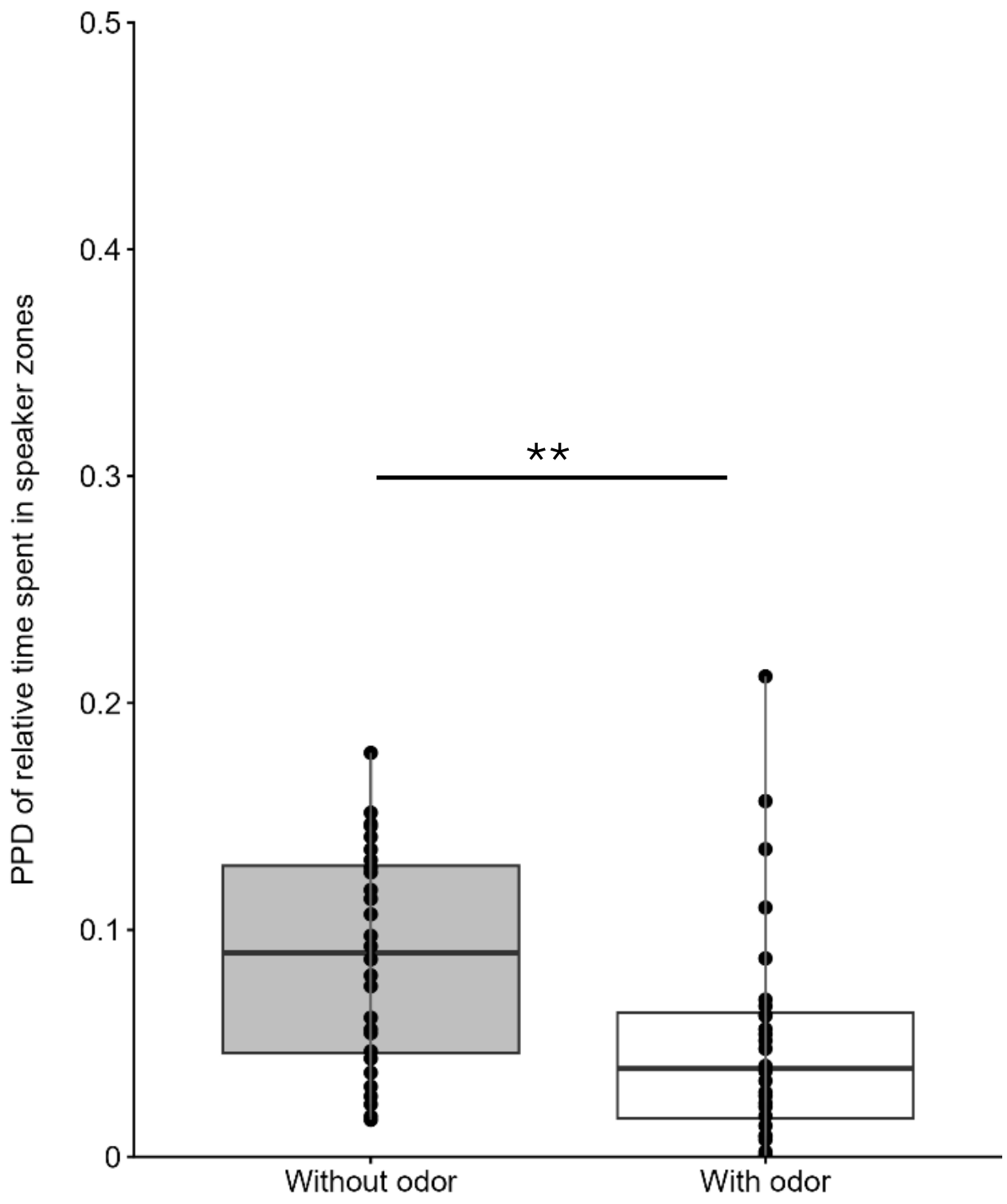

Supplementary Figure S7: Odor effect on PPD in experiment 3. Boxplots of odor effect on the phase preference difference (PPD) of relative time spent in the speaker zones. Boxplots show the median, 25% and 75% quartiles and whiskers extending to the most extreme data points within 1.5x the interquartile range. Dots represent individual data points; $n_{without}$=14, n_{with}=14; **=p≤0.01.

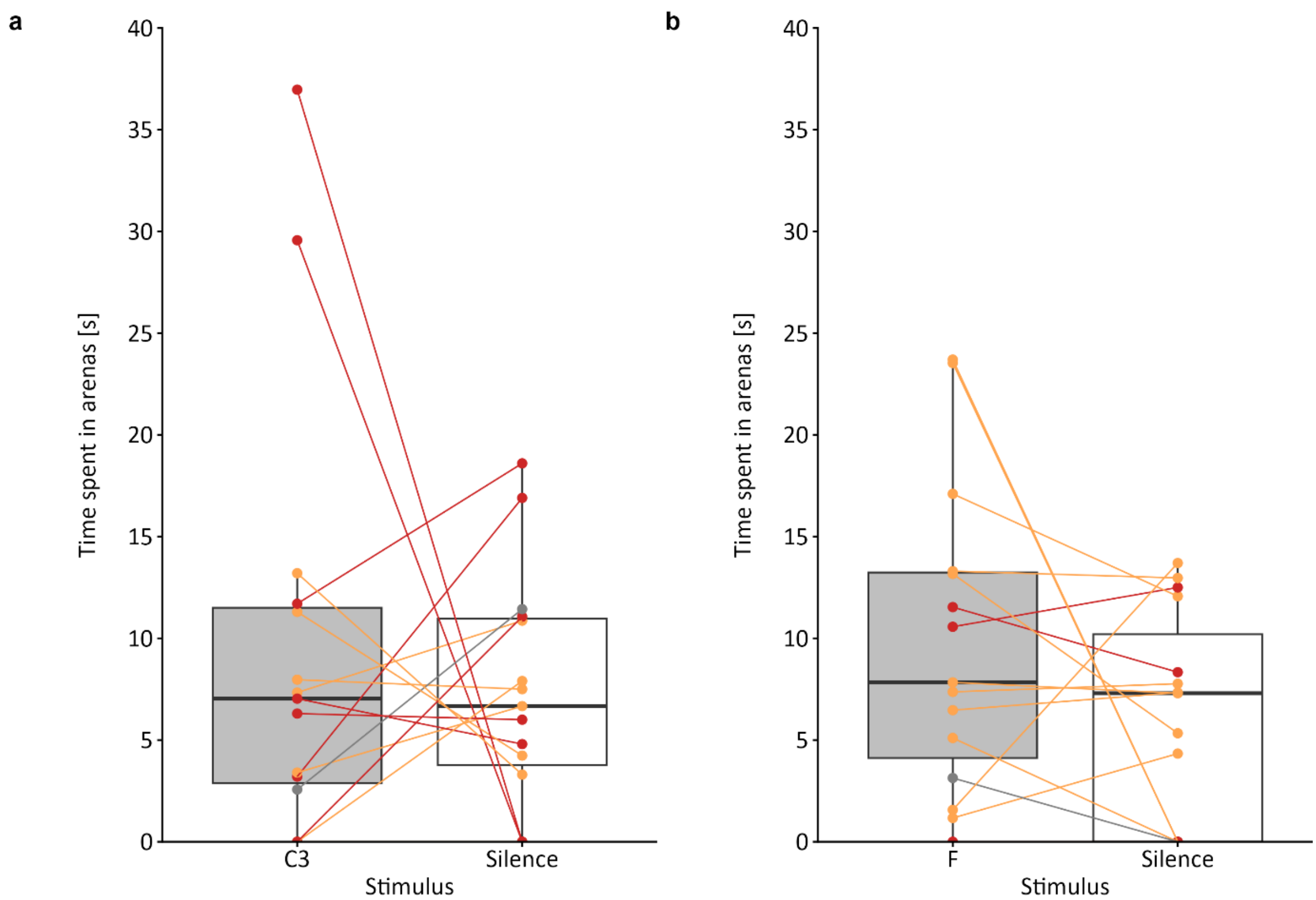

Supplementary Figure S8: Females' behavioral responses in different estrous stages to playbacks (C3 or F) compared to silence (experiment 2). Boxplots of time [s] spent in the arenas for a) C3 vs. silence and b) F vs. silence. Boxplots show the median, 25% and 75% quartiles and whiskers extending to the most extreme data points within 1.5x the interquartile range. Dots represent individual data points. The same individuals are connected by lines. Line and dot colors represent different estrous stages: red=estrus, yellow=non-estrus, grey=no cells; n_{c3}=15, n_{flat}=15.

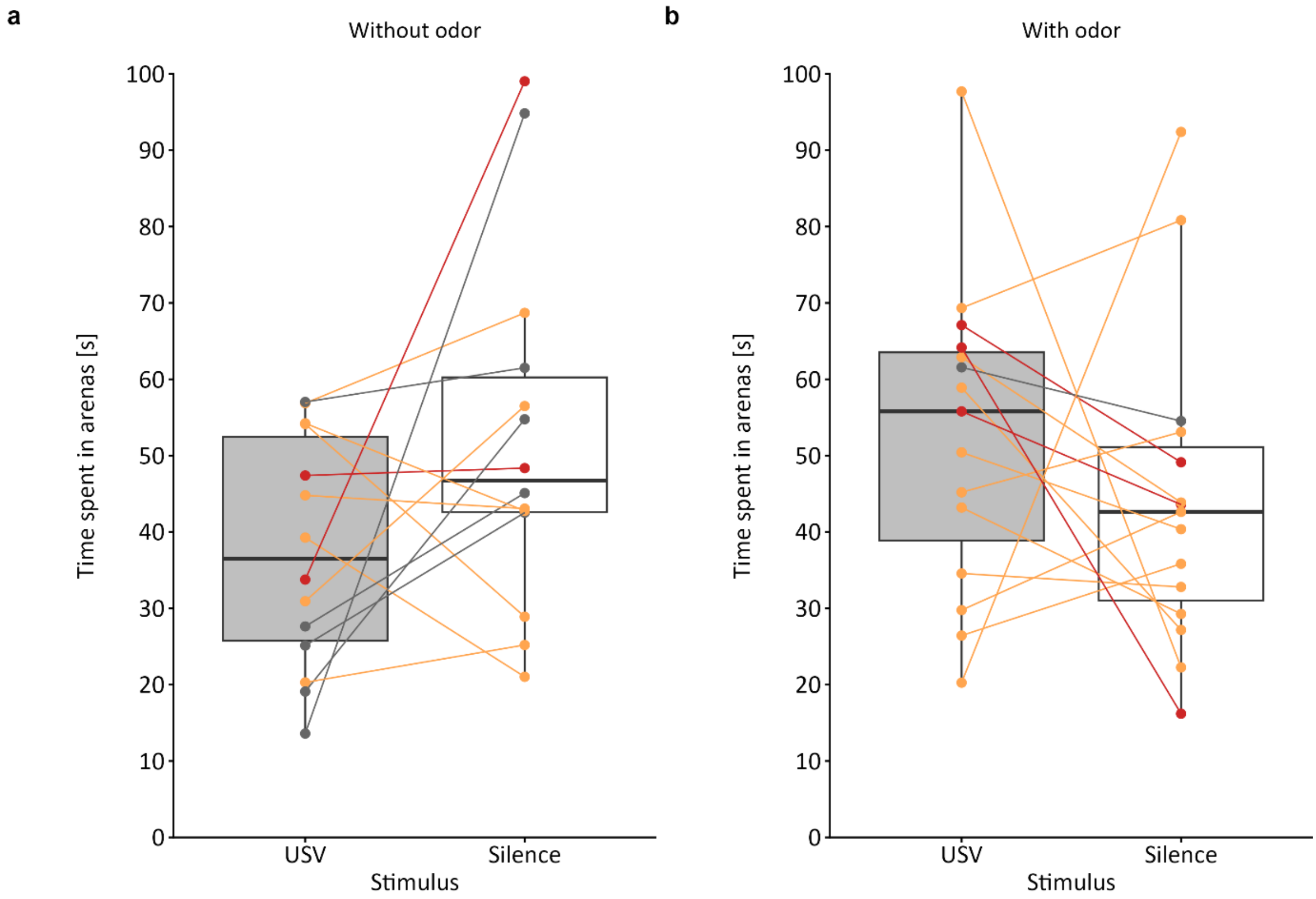

Supplementary Figure S9: Females' behavioral responses in different estrous stages to more natural male USV sequences compared to silence with and without male odor. Boxplots of time [s] spent in the arenas a) without and b) with odor. Boxplots show the median, 25% and 75% quartiles and whiskers extending to the most extreme data points within 1.5x the interquartile range. Dots represent individual data points. The same individuals are connected by lines. Line and dot colors represent different estrous stages: red=estrus, yellow=non-estrus, grey=no cells; $n_{without}$=14, n_{with}=15.

Supplementary tables:

Supplementary Table S1: Results of linear mixed-effects models (using Satterthwaite approximation) investigating effects of playback type (F and C3) on variables measured in the speaker zones and arenas of the Y-maze in experiment 1.

Predictor	Estimate	SE	df	t	p
Speaker zone					
**Duration**					
(Intercept)	0.09	0.25	32	0.37	0.717
Playback type	-0.18	0.35	32	-0.52	0.609
**Entries**					
(Intercept)	2	0.25	32	8.03	3.64×10^{-9}
Playback type	-0.12	0.35	32	-0.33	0.741
Arena					
**Duration**					
(Intercept)	0.08	0.25	32	0.33	0.746
Playback type	-0.16	0.35	32	-0.46	0.648
**Entries**					
(Intercept)	1.94	0.22	27.106	8.65	2.82×10^{-9}
Playback type	-0.12	0.24	16	-0.49	0.632
**Latency to first visit**					
(Intercept)	0.23	0.24	32	0.98	0.336
Playback type	-0.47	0.34	32	-1.38	0.176

Supplementary Table S2: Results of linear mixed-effects models (using Satterthwaite approximation) investigating the effects of testing day (day 1-3) and playback type (F and C3) on the duration in the speaker zones and arenas of the Y-maze in experiment 1.

Predictor	Estimate	SE	df	t	p
Day effect on duration					
**Speaker zone**					
(Intercept)	-0.38	0.43	28	-0.89	0.381
Playback type (F)	0.97	0.61	28	1.6	0.122
Day 2	1.08	0.58	28	1.85	0.075
Day 3	0.26	0.58	28	0.45	0.658
Playback type (F):Day2	-1.97	0.82	28	-2.4	0.024
Playback type (F):Day3	-1.28	0.82	28	-1.56	0.13
**Arena**					
(Intercept)	1.87	0.3	27.964	6.12	1.34×10^{-6}
Playback type (F)	0.73	0.42	14	1.74	0.105
Day 2	0.62	0.41	27.964	1.51	0.143
Day 3	0.22	0.41	27.964	0.54	0.594
Playback type (F):Day2	-1.39	0.57	14	-2.42	0.03
Playback type (F):Day3	-1.07	0.57	14	-1.86	0.084

Supplementary Table S3: Results of linear mixed-effects models of the effects of phase (familiarization and playback phase) and playback type (F and C3) on the phase preference difference (PPD) in the speaker zones and arenas of the Y-maze in experiment 1. **Total:** comparing the whole familiarization phase with the whole playback phase. **40 s:** comparing last 40 s of familiarization phase with the playback phase (also 40 s). Model coefficients (fixed effects) are shown on the right. F-tests of fixed effects (Satterthwaite method) are shown on the left.

Predictor	Num df	Den df	F	p	η^2	Estimate	SE	df	t	p
Speaker zone										
**Total**										
(Intercept)						-0.32	0.29	31	-1.11	0.274
Phase	1	31	5.90	0.021	0.16	0.82	0.34	31	2.43	0.021
Playback type	1	31	1.26	0.270	0.04	-0.37	0.33	31	-1.12	0.271
**40 s**										
(Intercept)						0.22	0.29	30.858	0.76	0.453
Phase	1	21.793	1.99	0.172	0.08	-0.42	0.3	21.793	-1.41	0.172
Playback type	1	15.172	0.02	0.904	0.000997	0.03	0.27	15.172	0.12	0.904
Arena										
**Total**										
(Intercept)						-0.30	0.35	30.841	-0.88	0.388
Phase	1	29.813	1.78	0.192	0.06	0.51	0.38	29.813	1.34	0.192
Playback type	1	14.136	0.10	0.751	0.00734	-0.11	0.34	14.137	-0.32	0.751
**40 s**										
(Intercept)						-0.33	0.28	30.957	-1.18	0.249
Phase	1	20.324	0.21	0.652	0.01	0.11	0.31	20.507	0.35	0.733
Playback type	1	15.277	3.08	0.099	0.17	0.56	0.3	15.282	1.91	0.075

Supplementary Table S4: Results of linear mixed-effects models (using Satterthwaite approximation) investigating effects of stimulus type (USV and silence) and playback type (F and C3) on variables measured in the speaker zones and arenas of the Y-maze in experiment 2.

Predictor	Estimate	SE	df	t	p
Speaker zone					
**Duration**					
(Intercept)	2.09	0.3	57	7.03	2.85×10^{-9}
Stimulus type	-0.42	0.34	57	-1.23	0.226
Playback type	0.01	0.34	57	0.02	0.987
**Entries**					
(Intercept)	-0.01	0.25	37.8	-0.06	0.954
Stimulus type	-0.01	0.19	29	-0.06	0.952
Playback type	0.04	0.32	28	0.13	0.901
Arena					
**Duration**					
(Intercept)	10.04	1.79	5.564	5.62	0.002
Stimulus type	-3.25	2.03	52	-1.6	0.116
Playback type	-0.02	2.04	41.609	-0.01	0.994
**Entries**					
(Intercept)	0.13	0.23	43.697	0.56	0.576
Stimulus type	-0.31	0.23	29	-1.39	0.176
Playback type	0.05	0.29	28	0.17	0.866
**Latency to first visit**					
(Intercept)	13.91	2.47	45	5.64	1.05×10^{-6}
Stimulus type	-0.47	2.86	45	-0.16	0.871
Playback type	4.14	2.85	45	1.45	0.153

Supplementary Table S5: Results of linear mixed-effects models of the effects of phase (familiarization and playback phase), stimulus type (USV and silence) and playback type (F and C3) on the phase preference difference (PPD) in the speaker zones and arenas of the Y-maze in experiment 2. **Total:** comparing the whole familiarization phase with the whole playback phase. **40 s:** comparing last 40 s of familiarization phase with the playback phase (also 40 s). Model coefficients (fixed effects) are shown on the right. F-tests of fixed effects (Satterthwaite method) are shown on the left.

Predictor	Num df	Den df	F	p	η^2	Estimate	SE	df	t	p
Speaker zone										
**Total**										
(Intercept)						-0.17	0.31	9.53	-0.54	0.599
Phase	1	48.541	1.12	0.295	0.02	0.29	0.27	48.541	1.06	0.295
Stimulus type	1	47.153	0.13	0.712	0.00282	-0.09	0.26	47.153	-0.37	0.717
Playback type	1	48.195	0.21	0.647	0.00439	0.12	0.27	48.195	0.46	0.647
**40 s**										
(Intercept)						0.18	0.32	2.239	0.57	0.62
Phase	1	34.6	1.91	0.176	0.05	-0.41	0.3	34.6	-1.38	0.176
Stimulus type	1	21.451	0.03	0.86	0.00148	-0.04	0.25	21.451	-0.18	0.86
Playback type	1	20.464	0.01	0.934	0.000339	-0.03	0.33	20.464	-0.08	0.934
Arena										
**Total**										
(Intercept)						-0.35	0.32	8.008	-1.09	0.306
Phase	1	45.887	0.97	0.331	0.02	0.25	0.26	45.887	0.98	0.330
Stimulus type	1	25.563	0.32	0.578	0.01	0.14	0.25	25.563	0.56	0.578
Playback type	1	22.015	1.73	0.202	0.07	0.33	0.25	22.015	1.32	0.202
**40 s**										
(Intercept)						0.15	0.4	1.718	0.38	0.747
Phase	1	39.377	0.03	0.862	0.000781	-0.05	0.31	39.377	-0.18	0.862
Stimulus type	1	21.375	2.04	0.168	0.09	-0.38	0.27	21.375	-1.43	0.167
Playback type	1	20.659	0.96	0.339	0.04	0.3	0.31	20.659	0.98	0.339

Supplementary Table S6: Results of linear mixed-effects models (using Satterthwaite approximation) investigating effects of stimulus type (USV and silence) and odor (with and without) on variables measured in the speaker zones and arenas of the Y-maze in experiment 3.

Predictor	Estimate	SE	df	t	p
Speaker zone					
**Duration**					
(Intercept)	0.7	0.37	1.273	1.87	0.27
Stimulus type	-0.27	0.27	37.963	-0.98	0.333
Odor	-0.79	0.31	1.606	-2.59	0.152
**Entries**					
(Intercept)	0.17	0.28	15.886	0.61	0.549
Stimulus type	-0.26	0.18	27.997	-1.42	0.166
Odor	-0.12	0.29	21.771	-0.41	0.684
Arena					
**Duration**					
(Intercept)	46.72	5.68	3.319	8.23	0.003
Stimulus type	-2.8	6	41.986	-0.47	0.643
Odor	2.4	6.15	41.993	0.39	0.699
**Entries**					
(Intercept)	-0.16	0.27	22.759	-0.6	0.554
Stimulus type	0.08	0.13	28	0.6	0.551
Odor	0.22	0.35	23.334	0.63	0.532
**Latency to first visit**					
(Intercept)	-0.02	0.27	27.033	-0.06	0.955
Stimulus type	-0.25	0.27	36.89	-0.93	0.357
Odor	0.19	0.28	42.768	0.66	0.514

Supplementary Table S7: Results of linear mixed-effects models of the effects of phase (familiarization and playback phase), stimulus type (USV and silence) and odor (with and without) on the phase preference difference (PPD) of the familiarization and playback phase in total in the speaker zones and arenas of the Y-maze in experiment 3. Model coefficients (fixed effects) are shown on the right. F-tests of fixed effects (Satterthwaite method) are shown on the left.

Predictor	Num df	Den df	F	p	η^2	Estimate	SE	df	t	p
**Speaker zone**										
(Intercept)						0.07	0.02	52	4.29	7.71×10^{-5}
Phase	1	52	1.33	0.255	0.02	0.02	0.01	52	1.15	0.255
Stimulus type	1	52	0.94	0.337	0.02	0.01	0.01	52	0.97	0.337
Odor	1	52	7.51	0.008	0.13	-0.04	0.01	52	-2.74	0.008
**Arena**										
(Intercept)						0.25	0.06	40.892	4.09	0.0002
Phase	1	33.374	3.36	0.076	0.09	0.08	0.05	33.374	1.83	0.076
Stimulus type	1	22.126	0.22	0.640	0.01	0.02	0.03	22.126	0.47	0.64
Odor	1	20.971	0.003	0.959	0.000126	-0.002	0.05	20.971	-0.05	0.959
